# ﻿The Trichoptera of Panama. XIX. Additions to and a review of the genus *Leucotrichia* (Trichoptera, Hydroptilidae) in Panama

**DOI:** 10.3897/zookeys.1111.77371

**Published:** 2022-07-11

**Authors:** Robin E. Thomson, Brian J. Armitage, Steven C. Harris

**Affiliations:** 1 University of Minnesota, Department of Entomology, 1980 Folwell Ave., St. Paul, MN 55108, USA University of Minnesota St. Paul United States of America; 2 Museo de Peces de Agua Dulce e Invertebrados, Universidad Autónoma de Chiriquí, David, Panama Universidad Autónoma de Chiriquí David Panama; 3 Department of Biology and Geosciences, Clarion University, Clarion, PA, USA Clarion University Clarion United States of America

**Keywords:** Caddisflies, collection methods, Leucotrichiinae, new species, species coexistence

## Abstract

Prior to 2016, three species of caddisflies in the genus *Leucotrichia* (Trichoptera: Hydroptilidae) were known from Panama. Subsequently, one new species and four new country records were added to Panama’s fauna. Herein, four new species are described (*Leucotrichiacortadera***sp. nov.**, *L.holzenthali***sp. nov.**, *L.luma***sp. nov.**, *L.ruiteri***sp. nov.**) and two new country records added for Panama (*L.botosaneanui* Flint, 1996, *L.hispida* Thomson & Holzenthal, 2015). The resulting total of 14 species makes Panama the most species-rich country for this genus. Panama’s species assemblage is most similar to Costa Rica and Mexico. However, the similarities among faunas in all these countries is very low (< 35%). Thus, more new country records are possible with additional collecting. Recent collections (2015–2021) of new caddisfly species and country records in this genus were effected primarily by use of Malaise traps. Our collections also evidenced multiple species from the same collecting site, with seven species each found in both lowland and mid-altitude sites. Investigation of the distribution of *Leucotrichia* species with altitude reveals a preference by several species for higher altitude locations. Additional Malaise trap collections over extended time periods are needed to verify the validity of all observations and preliminary conclusions made to date.

## ﻿Introduction

The genus *Leucotrichia* Mosely, 1934 is in the microcaddisfly family Hydroptilidae and subfamily Leucotrichiinae. Known only in the New World, species of this genus are found throughout the continental United States, Central America, South America, and much of the Caribbean (Thomson and Holzenthal 2015). The background and development of *Leucotrichia* as a genus, its relationships with other Leucotrichiinae genera, and the composition and relationships within the genus can best be understood by referencing Flint (1970), Marshall (1979), Oláh and Johanson (2011), Thomson and Holzenthal (2012, 2015), Santos et al. (2016), and Holzenthal and Calor (2017). The latter publication lists 44 extant species and one fossil species, *Leucotrichiaadela* Wells & Wichard, 1989 from Dominican amber. Since then, one new species has been added to the genus (Thomson and Armitage 2021).

The larval stages are dorsoventrally depressed and occupy lotic-erosional habitats. The first four instars are free-living, with these stages completed fairly rapidly (less than two weeks). This is followed by a fifth instar that builds an elliptical, flattened case, usually found firmly affixed to large rocks and boulders (Wiggins 1996a). These silken cases are purse-like and have round openings at either end. During pupation, the openings are sealed, with one subsequently cut open for egress by the pharate adult. Often cases are repaired and reused by subsequent generations (Wiggins 1996b). The larval diet of *Leucotrichia* species consist of periphyton and fine particulate organic matter, and they have been categorized as both scrapers and collector-gatherers. Adults can be collected in UV light traps or, more commonly, by sweeping streamside vegetation (Holzenthal and Calor 2017). We have found Malaise traps to be most productive.

*Leucotrichiaviridis* Flint, 1967 was the first species of this genus recorded from Panama (Flint 1970). In that same publication, *L.chiriquiensis* Flint, 1970 and *L.fairchildi* Flint, 1970 became the first two species originally described from Panama. These three species remained the extent of our knowledge for this genus in Panama until Armitage et al. (2016) added *L.melleopicta* Mosely, 1934, the nominate species for the genus, which was described from Mexico. Subsequently, new additions were made: *L.extraordinaria* Bueno-Soria, Santiago-Fragoso & Barba-Álvarez, 2001 and *L.mutica* Flint, 1991 by Armitage et al. (2018); *L.rhomba* Thomson & Holzenthal, 2015 by Harris and Armitage (2019); and, recently a new species, *L.cultrata* Thomson & Armitage, 2021. Herein, we add four new species and two new country records, bringing the total number of species known from Panama to 14. Further, based on our discoveries for this genus in Panama, we have amassed sufficient data to discuss and perhaps speculate on the topics of potential diversity, coexistence, altitudinal distribution, and geographical affinities.

## ﻿Materials and methods

Single, overnight collections were made using UV light traps (Calor and Mariano 2012). Multiple-day collections were made employing Malaise traps over four or more 24-hour periods. Collection locations are presented in Fig. 17.

Morphological terminology used for male genitalia generally follows that of Marshall (1979), as mirrored by Thomson and Holzenthal (2015). For simplicity, paired structures are discussed in the singular. Procedures for specimen preparation followed those explained in detail by Blahnik et al. (2007). For specimen examination and illustration, cleared genitalia were placed in a watch glass with glycerin and cotton. Genitalia were examined with an Olympus BX43 compound microscope at 250–500 × magnification. Structures were traced in pencil with the use of a camera lucida (drawing tube) mounted on the microscope. Pencil sketches were scanned (Fujitsu ScanScap S1500M scanner) and were then edited and digitally inked in Adobe Photoshop and Illustrator (CS5.1). Electronic “drawing” was completed with the aid of a graphics tablet (Bamboo Pen, Wacom Co., Ltd.). Species descriptions were constructed using the program DELTA (Dallwitz et al. 2016).

All specimens included in this publication are stored in 80% alcohol. Holotypes of the species described are deposited in the Universidad de Panamá Museo de Invertebrados (**MIUP**). Other specimens are deposited in the University of Minnesota Insect Collection (**UMSP**), the Museo de Peces de Agua Dulce e Invertebrados (**MUPADI**) of the Universidad Autónoma de Chiriquí (**UNACHI**), or the Colección Zoológica Dr. Eustorgio Méndez (**COZEM**) of the Instituto Conmemorativo Gorgas de Estudios de la Salud (Gorgas Institute).

## ﻿Results

The distribution of species among the 19 unique locations within Panama wherein the genus *Leucotrichia* has been collected to date is presented in Table 1, which complements Fig. 17. We have sampled, to varying degrees, 16 of the 52 major cuencas (watersheds) in Panama and found species of *Leucotrichia* in eight of these (Fig. 17; Table 2). In addition, we have found them in all five of the administrative units (of a total of 14: 10 provinces and four comarcas) which have been sampled to date.

**Table 1.** Species associated with each collected stream. See Fig. 17 for locations and Table 2 for watershed (cuenca) information.

Label	Cuenca	Stream	Species
**1**	**102**	**Rio Candela-Finca Felix**	*Leucotrichiachirquiensis*
			*Leucotrichiahispida*
			*Leucotrichiaruiteri*
**2**	**102**	**Quebrada Norte**	*Leucotrichiachirquiensis*
			*Leucotrichiacultrata*
**3**	**102**	**Afl. Rio Chiriqui Viejo**	*Leucotrichiahispida*
**4**	**102**	**Rio Chiriqui Viejo**	*Leucotrichiahispida*
**5**	**106**	**Rio Chirigagua**	*Leucotrichiaextraordinaria*
**6**	**108**	**Quebrada del Guayabo**	*Leucotrichiahispida*
**7**	**108**	**Quebrada Grande**	*Leucotrichiaextraordinaria*
			*Leucotrichiamutica*
**8**	**108**	**Quebrada Jaramillo**	*Leucotrichiabotosaneanui*
			*Leucotrichiacortadera*
			*Leucotrichiacultrata*
			*Leucotrichiaextraordinaria*
			*Leucotrichiamelleopicta*
			*Leucotrichiarhomba*
			*Leucotrichiaruiteri*
**9**	**108**	**Rio Majagua**	*Leucotrichiacortadera*
			*Leucotrichiacultrata*
			*Leucotrichiaextraordinaria*
			*Leucotrichiamelleopicta*
			*Leucotrichiarhomba*
			*Leucotrichiaviridis*
**10**	**93**	**Quebrada Martinez**	*Leucotrichiafairchildi*
			*Leucotrichiamelleopicta*
			*Leucotrichiamutica*
**11**	**93**	**Quebrada Rambala**	*Leucotrichiacultrata*
			*Leucotrichiaextraordinaria*
			*Leucotrichiafairchildi*
			*Leucotrichiamelleopicta*
			*Leucotrichiamutica*
			*Leucotrichiarhomba*
			*Leucotrichiaviridis*
**12**	**97**	**Rio Llanito**	*Leucotrichiamelleopicta*
**13**	**97**	**Rio Piedra de Moler**	*Leucotrichiaextraordinaria*
			*Leucotrichiafairchildi*
			*Leucotrichiaholzenthali*
			*Leucotrichiamelleopicta*
**14**	**97**	**Rio Calovebora**	*Leucotrichiacultrata*
			*Leucotrichiaextraordinaria*
**15**	**97**	**afl. Rio Calovebora**	*Leucotrichiaextraordinaria*
			*Leucotrichiamelleopicta*
			*Leucotrichiamutica*
**16**	**132**	**Rio Mulaba, 2do Brazo**	*Leucotrichiaextraordinaria*
			*Leucotrichiamelleopicta*
			*Leucotrichiarhomba*
			*Leucotrichiaruiteri*
**17**	**132**	**Rio Mulaba, afl. 1er Brazo**	*Leucotrichiacultrata*
			*Leucotrichiamelleopicta*
			*Leucotrichiaviridis*
**18**	**115**	**Rio Chileno**	*Leucotrichiacultrata*
			*Leucotrichialuma*
**19**	**138**	**Rio Sajalice**	*Leucotrichialuma*

**Table 2.** Major watersheds in which *Leucotrichia* species have been collected.

Cuenca No.	Major River	Drainage Area (km^2^)	Receiving Body
**93**	Guariviara	2121	Caribbean Sea
**97**	Calovébora	485	Caribbean Sea
**102**	Chiriqui Viejo	1376	Pacific Ocean
**106**	Chico	593	Pacific Ocean
**108**	Chiriqui	1905	Pacific Ocean
**115**	Chagres	3338	Caribbean Sea
**132**	Santa Maria	3326	Pacific Ocean
**138**	Chame	1476	Pacific Ocean

In Table 2, we provide additional information about the major watersheds (cuencas) in which *Leucotrichia* has been collected in Panama. The number of unique locations in which each species has been found to date is presented in Table 3. In Table 4, we present the species which potentially could be found in Panama as new country records. These species were selected by targeting countries in which shared species currently occur. The distribution by altitude for each species known from Panama is presented in Fig. 18. Whereas, most species have been collected in a somewhat broad spectrum of altitudes, particularly in the low to mid-altitude range, at least a few appear to be restricted to higher altitudes. We should note that Fig. 18 was constructed from unique records, single records from each sample location/stream, and does not reflect multiple records from the same sites over a single or multiple year period.

**Table 3.** Number of unique streams (*max* = 19) in which each *Leucotrichia* species was found.

Species	No. of Streams
*Leucotrichiaextraordinaria*	**9**
*Leucotrichiamelleopicta*	**9**
*Leucotrichiacultrata*	**7**
*Leucotrichiahispida*	**4**
*Leucotrichiamutica*	**4**
*Leucotrichiarhomba*	**4**
*Leucotrichiafairchildi*	**3**
*Leucotrichiaruiteri*	**3**
*Leucotrichiaviridis*	**3**
*Leucotrichiachirquiensis*	**2**
*Leucotrichiacortadera*	**2**
*Leucotrichialuma*	**2**
*Leucotrichiabotosaneanui*	**1**
*Leucotrichiaholzenthali*	**1**
**Means**:	**2.84 species stream^-1^**
	**3.86 streams species^-1^**

**Table 4.** Species of *Leucotrichia* which could potentially be found in Panama, based on the current range distributions of its species.

Species	Current distribution
***L.melleopicta* group**	
*Leucotrichiaangelinae* Thomson & Holzenthal, 2015	**Venezuela**
*Leucotrichiaayura* Flint, 1991	**Colombia**
*Leucotrichiabrochophora* Flint, 1991	**Colombia**
*Leucotrichiadenticulata* Thomson & Holzenthal, 2015	**Mexico**
*Leucotrichiadianeae* Thomson & Holzenthal, 2015	**Costa Rica**
*Leucotrichiadinamica* Bueno-Soria, 2010	**Mexico**
*Leucotrichiaforrota* Oláh & Johanson, 2011	**Ecuador, Peru**
*Leucotrichiafulminea* Thomson & Holzenthal, 2015	**Ecuador**
*Leuoctrichiainflaticornis* Botosaneanu, in Botosaneanu and Alkins-Koo 1993	**Trinidad**
*Leucotrichiainops* Flint, 1991	**Colombia, Ecuador**
*Leucotrichiainterrupta* Flint, 1991	**Colombia**
*Leucotrichiakateae* Thomson & Holzenthal, 2015	**Venezuela**
*Leucotrichialimpia* Ross, 1944	**Costa Rica, Mexico, U.S.A.**
*Leucotrichiapadera* Flint, 1991	**Colombia**
*Leucotrichiapectinata* Thomson & Holzenthal, 2015	**Ecuador**
*Leucotrichiarepanda* Thomson & Holzenthal, 2015	**Venezuela**
*Leucotrichiariostoumae* Thomson & Holzenthal, 2015	**Ecuador**
*Leucotrichiasidneyi* Thomson & Holzenthal, 2015	**Venezuela**
*Leucotrichiatapantia* Thomson & Holzenthal, 2015	**Costa Rica**
*Leucotrichiatermitiformis* Botosaneanu, in Botosaneanu and Alkins-Koo 1993	**Trinidad**
*Leucotrichiatritoven* Flint, 1996	**Guyana, Tobago, Trinidad, Venzuela**
*Leucotrichiazopilote* (Holzenthal & Harris, 1999)	**Costa Rica**
***L.pictipes* group**	
*Leucotrichiapictipes* Banks, 1911	**Mexico, U.S.A.**
*Leucotrichiaimitator* Flint, 1970	**Costa Rica, Guatemala, Mexico**
*Leucotrichiasarita* Ross, 1944	**Costa Rica, El Salvador, Grenada, Guatemala, Mexico, Nicaragua, U.S.A.**

A perusal of the country distributions in the species accounts given below is not overly informative. Only one species, *L.fairchildi*, is somewhat widespread. Six species occupy portions of a distributional axis from Mexico southeast to northern South America (Colombia, Venezuela). Two other species have a distribution which involves (*L.botosaneanui*) or includes (*L.fairchildi*) Trinidad and Panama, which is orthogonal to this more common northwest/southeast axis. Six *Leucotrichia* species currently are endemic to Panama, including the four species described herein.

Confining ourselves primarily to countries with four or more species, we calculated similarity values and constructed a cluster diagram to show the relative affinity among seven Latin American countries (Fig. 19). Brazil, with three species, was included because of its role (Santos et al. 2016) in the evolution of the subfamily, tribe, and perhaps genus over geologic time. First, as expanded upon below, Brazil shares no species with other countries for this genus. The second result of the analysis is that none of the affinities among countries reach the 50% level. Costa Rica and Mexico approached that value, but fell short. Panama, currently with the most species of any country, is only ~ 35% similar to the combined diversity of Costa Rica and Mexico, and even less with the other countries presented herein.

### ﻿Taxonomy

#### Diagnosis of *Leucotrichia*

As expanded upon in the Discussion section, we think it is difficult at the present time to provide a definitive diagnosis for this genus based on adult characters. *Leucotrichia* is characterized by a prominent row of setae along the posterior margin of segment IX; a ventral process on abdominal segment VII in almost all species; inferior appendages simple, fused or not, bearing a dorsal spine in most species; and the subgenital plate with a ventral arm, accompanied by a dorsal arm in ca. half of the species.

With this equivocal diagnosis, we present fourteen species of *Leucotrichia* which we have found to date in Panama. Included are four new species to science and two first country records for Panama. Several of the species defined below bear morphological characters which are exceptions to those that served heretofore as diagnostic for this genus. Flint (1970), in his generic revision of this genus, identified characters which distinguish two species groups: *L.melleopicta* Group and *L.pictipes* Group. Our presentation of species below is organized within these two subgeneric groupings. Additional information about each species, including citations in which each species is referenced, can be found in Holzenthal and Calor (2017).

The plates for three species (*L.melleopicta*, *L.mutica*, and *L.rhomba*) were modified from Harris and Armitage (2019). We did this purposefully because these reflect study of numerous specimens from different parts of Panama, as well as exhibiting some small variations with those provided in Thomson and Holzenthal (2015). Drawings in the latter publication were sometimes based on a single individual, and not from Panama. We suggest researchers reference plates in both publications when identifying material from outside Panama. Further rationale and discussion of this subject is provided in the Remarks section under the species accounts below for the three taxa listed above.

**Figure 1.**
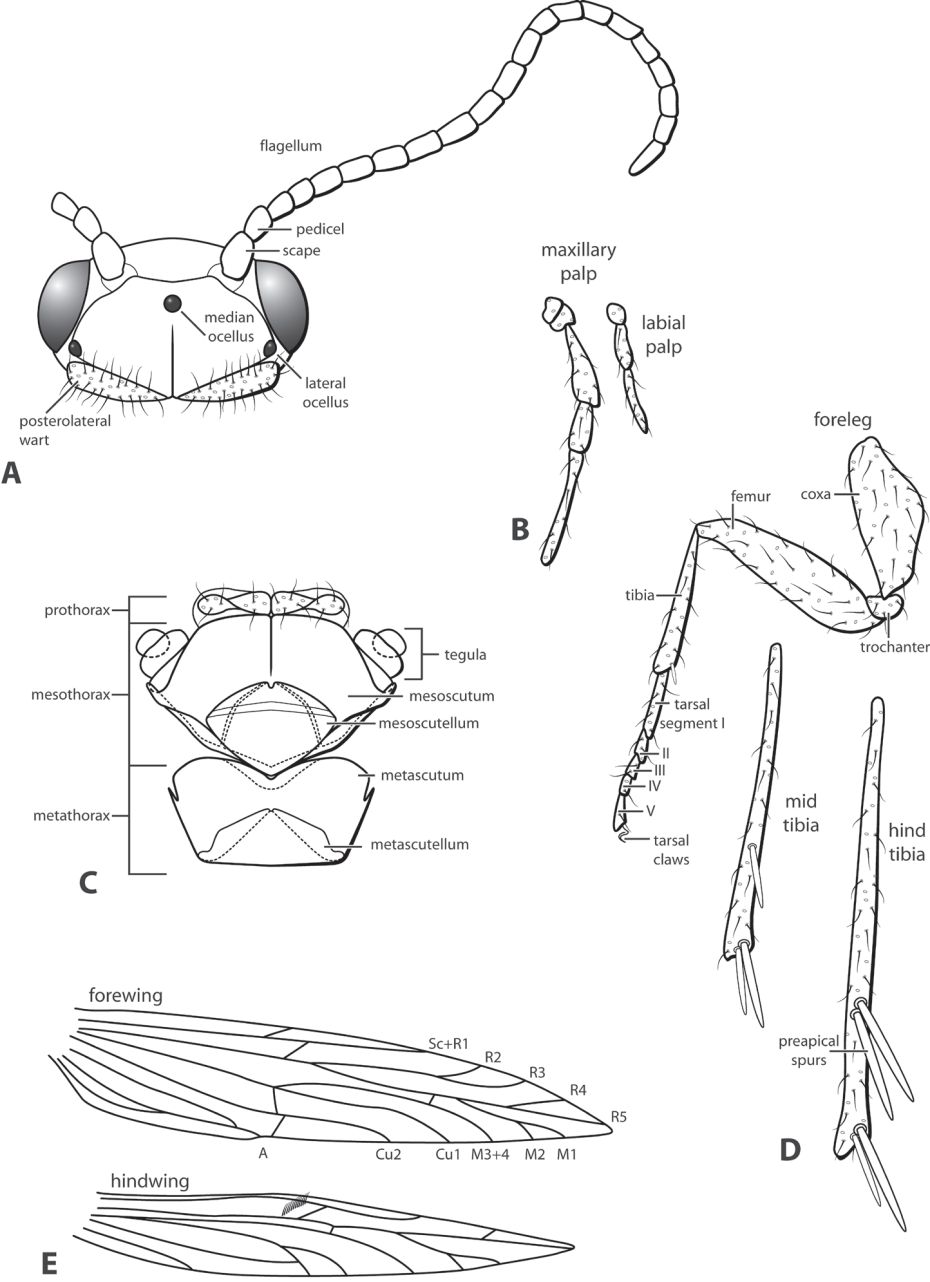
Unmodified *Leucotrichia***A** head and antennae, dorsal **B** palps **C** thorax, dorsal **D** legs and spur formula (1, 3, 4) **E** wings. Modified from Thomson and Holzenthal (2015).

**Figure 2.**
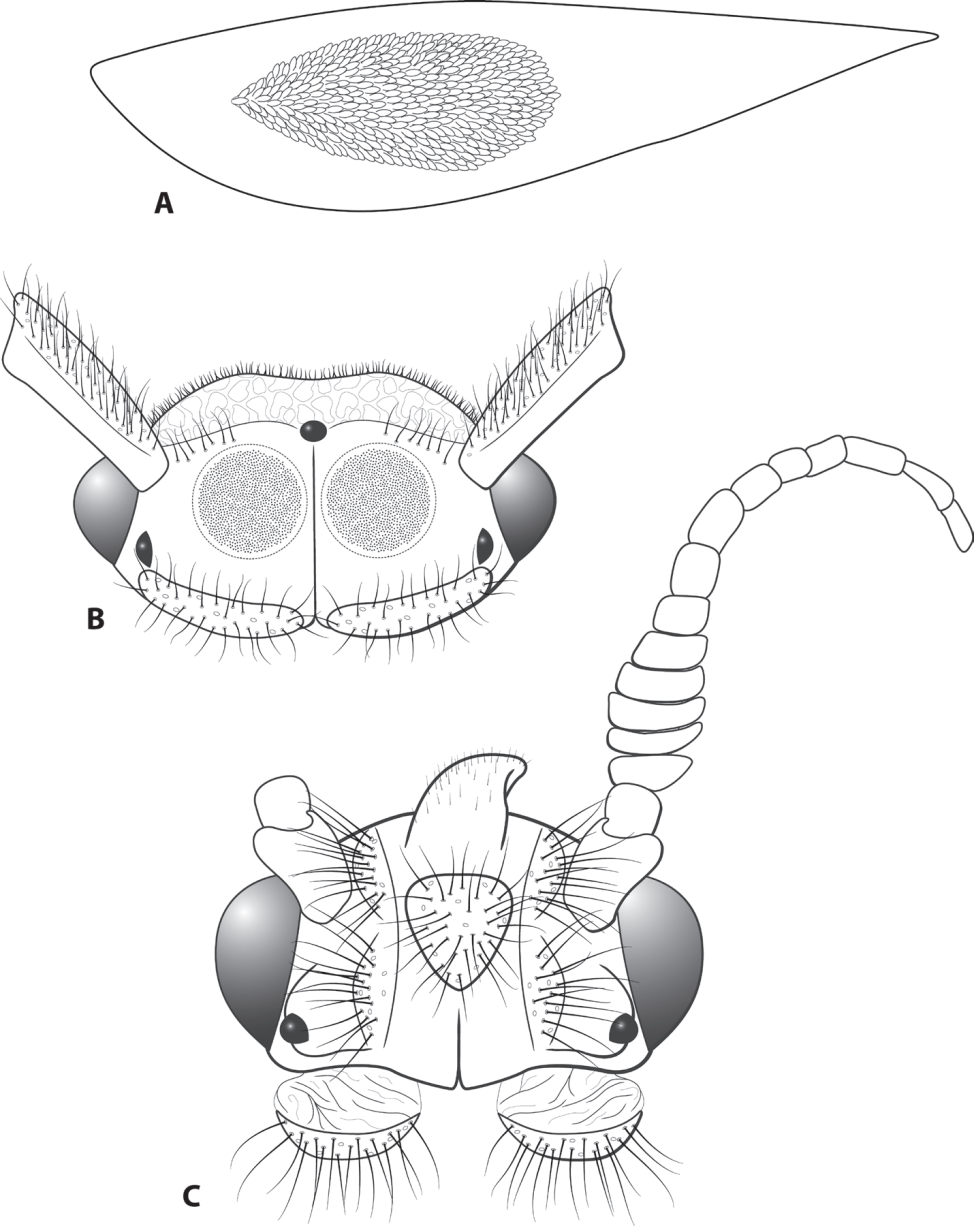
Modifications **A** Forewing, *Leucotrichiaruiteri* sp. nov. **B** head and scape, *Leucotrichiachiriquiensis* Flint, 1970 **C** head and antennae, *Leucotrichiafairchildi* Flint, 1970. Fig. 2B and 2C modified from Thomson and Holzenthal (2015).

General drawings for unmodified *Leucotrichia* body parts are given in Fig. 1. Examples of body parts which are modified in some species are presented in Fig. 2.

#### *Leucotrichiamelleopicta* group

##### ﻿ Leucotrichia botosaneanui

Taxon classificationAnimaliaTrichopteraHydroptilidae

Flint, 1996

03DC0D07-BD59-553A-B6C4-E88B6D7E5382

Fig. 3

###### Diagnosis.

**Figure 3.**
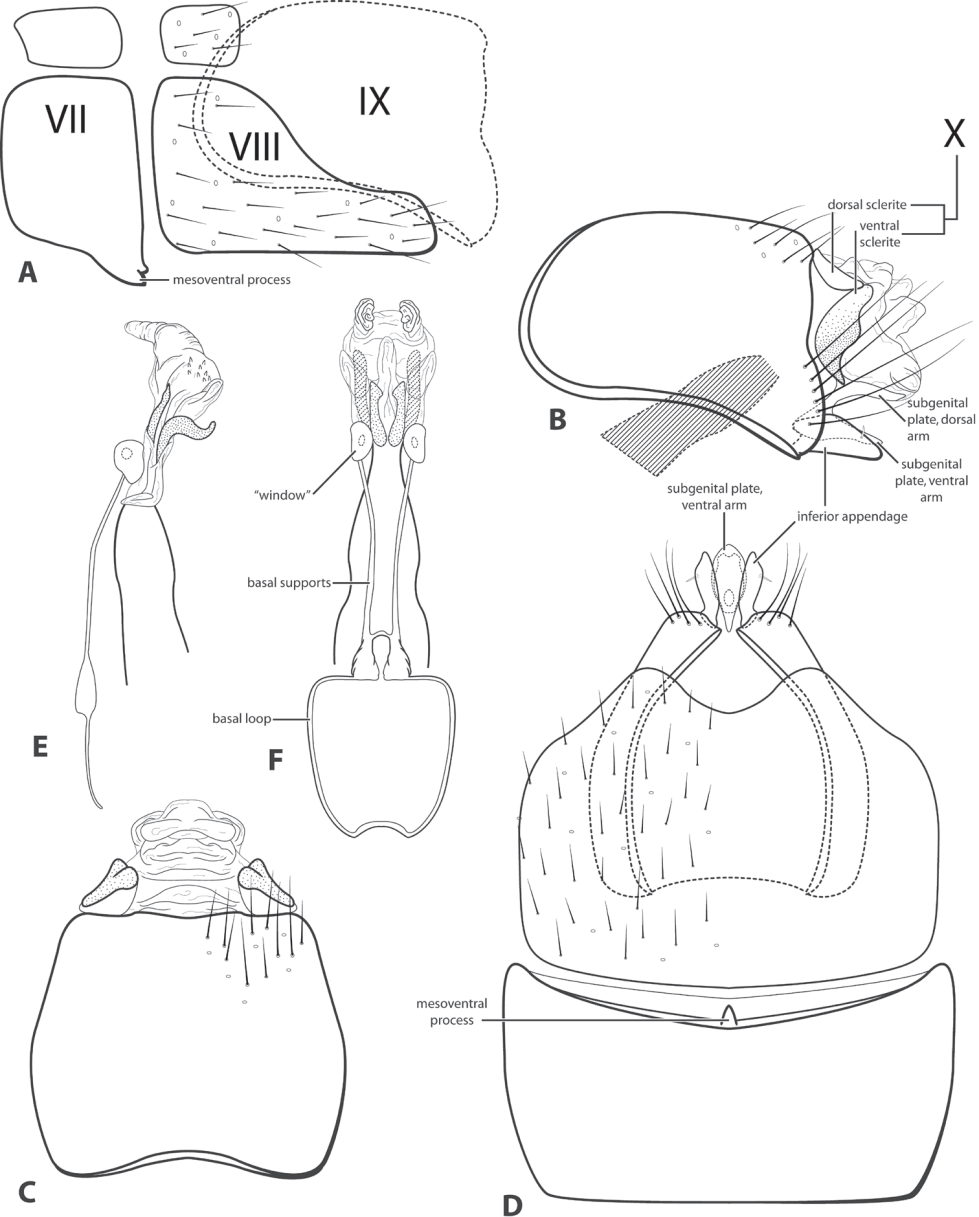
*Leucotrichiabotosaneanui* Flint, 1996. Male genitalia: **A** segments VII–VIII and segment IX margin, lateral **B** segments IX–X, lateral (base of phallus crosshatched) **C** segments IX–X, dorsal **D** segments VII–IX, ventral **E** phallus, lateral **F** phallus, dorsal. Modified from Thomson and Holzenthal (2015).

This species is similar to *L.chiriquiensis*, *L.hispida* Thomson & Holzenthal, 2015, and *L.viridis*, three species that also occur in Panama. The phallus of all four species has a similar appearance, due to the elongate basal supports of the midlength complex and the small pair of membranous, apical lobes (Fig. 3E, F). *Leucotrichiabotosaneanui* can be distinguished by the small, double-pointed mesoventral process on sternum VII, which is longer and much more prominent in the other species.

###### Material examined.

**Panama: Chiriqui Province** • 1 male; Cuenca 108; Boquete District; Quebrada Jaramillo; Finca Monterey; 8.7632°N, 82.41383°W; 1,214 m a.s.l.; 19–25 Apr. 2018; K. Collier, leg.; Malaise trap; in alcohol; MUPADI.

###### Distribution.

Panama, Trinidad, Tobago.

##### ﻿ Leucotrichia chiriquiensis

Taxon classificationAnimaliaTrichopteraHydroptilidae

Flint, 1970

A77EC3F6-5FEF-538A-8C50-5098568919EF

Figs 2B , 4

###### Diagnosis.

*Leucotrichiachiriquiensis* is most similar to *L.botosaneanui*, *L.hispida*, and *L.viridis*, based on a similar appearance of the phallus, as discussed under *L.botosaneanui*. This species can most swiftly be identified as distinct from the others based on the structural modifications of the antennae and head.

**Figure 4.**
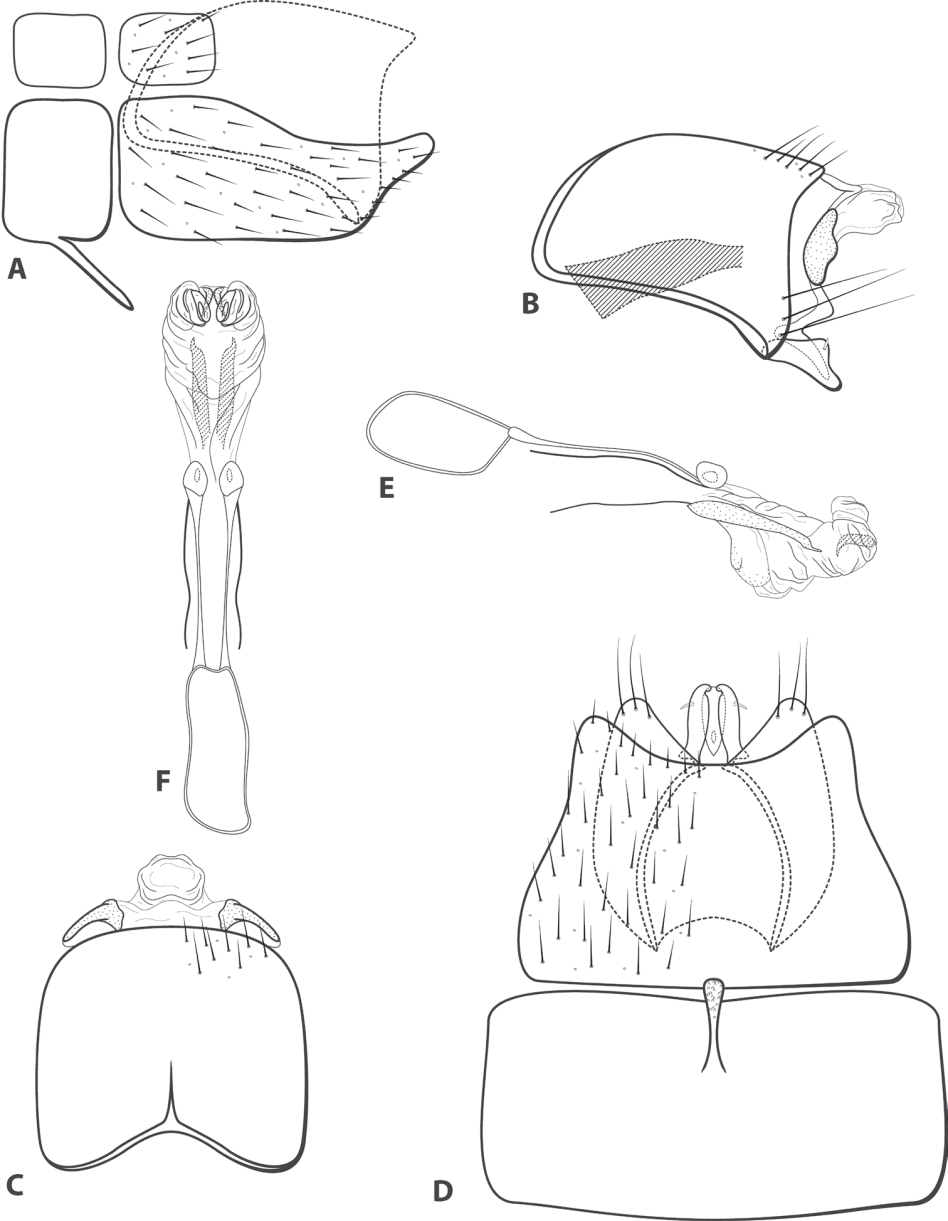
*Leucotrichiachiriquiensis* Flint, 1970. Male genitalia: **A** segments VII–VIII and segment IX margin, lateral **B** segments IX–X, lateral (base of phallus crosshatched) **C** segments IX–X, dorsal **D** segments VII–IX, ventral **E** phallus, lateral **F** phallus, dorsal. Modified from Thomson and Holzenthal (2015).

###### Material examined.

**Panama: Chiriqui Province** • 2 males; Cuenca 102; Renacimiento District; La Amistad International Park, Río Candela, Finca Felix, PSPSCB-PILA-C102-2017-021; 8.890557°N, 82.61201°W; 2,128 m a.s.l.; 25 Jan. 2015; C. Nieto, E. Pérez, A. Cornejo, leg.; UV light trap; in alcohol; COZEM • ibid., 2 males; Tierras Altas District; Mount Totumas Cloud Forest and Biological Reserve, Quebrada Norte; 8.873613°N, 82.690512°W; 1,709 m a.s.l.; 26 Apr.–10 May 2015; B. Armitage, T. Arefina-Armitage, leg.; Malaise trap; MUPADI • ibid., 5 males; 28 Jan.–2 Feb. 2018; J. Dietrich, leg.; Malaise trap; MUPADI • ibid., 3 males; 16–20 Feb. 2018; MUPADI • ibid., 1 male; 16–20 Mar. 2018; MUPADI • ibid., 1 male; 10–15 Jul. 2018; MUPADI • ibid., 2 males; 9–12 Sep. 2018; MUPADI • ibid., 2 males; 8–11 Nov. 2018; UMSP.

###### Distribution.

Panama.

##### ﻿ Leucotrichia cortadera sp. nov.

Taxon classificationAnimaliaTrichopteraHydroptilidae

BBACFDAB-10EA-57FE-BEDA-93ACF0CE36E6

http://zoobank.org/9EBB00CA-9C88-4BCC-9A07-8C5A250CF95E

Fig. 5

###### Type locality.

Panama: Chiriqui Province: Cuenca 108; Boquete District; Quebrada Jaramillo, Finca Monterey; 8.7632°N, 82.41383°W; 1,214 m a.s.l.

###### Type material.

***Holotype***: male, **Panama: Chiriqui Province**: Cuenca 108; Boquete District; Quebrada Jaramillo, Finca Monterey; 8.7632°N, 82.41383°W; 1,214 m a.s.l.; 16–20 Jun. 2018, K. Collier, leg.; Malaise trap; in alcohol; MIUP-001-T-2021. ***Paratype*: Panama: Chiriqui Province**: 1 male; Dolega District, Río Majagua, Banquito de Palmira, Potrerillos; 8.68093°N, 82.53276°W; 840 m a.s.l.; 19 Jul.–1 Aug. 2019, Y. Aguirre, T. Ríos, leg.; Malaise trap (M002); in alcohol; UMSP.

###### Diagnosis.

*Leucotrichiacortadera* sp. nov. is similar to *L.fulminea* Thomson & Holzenthal, 2015, a species endemic to Ecuador. Both species bear a pair of large, distinct sclerotized plates on the phallus apex. *Leucotrichiacortadera* can be separated by the small spines present on the dorsolateral surface of the phallus apex, which are absent in *L.fulminea*. Additionally, the inferior appendages are separate in *L.fulminea*, while they are fused in *L.cortadera*.

###### Description.

**Figure 5.**
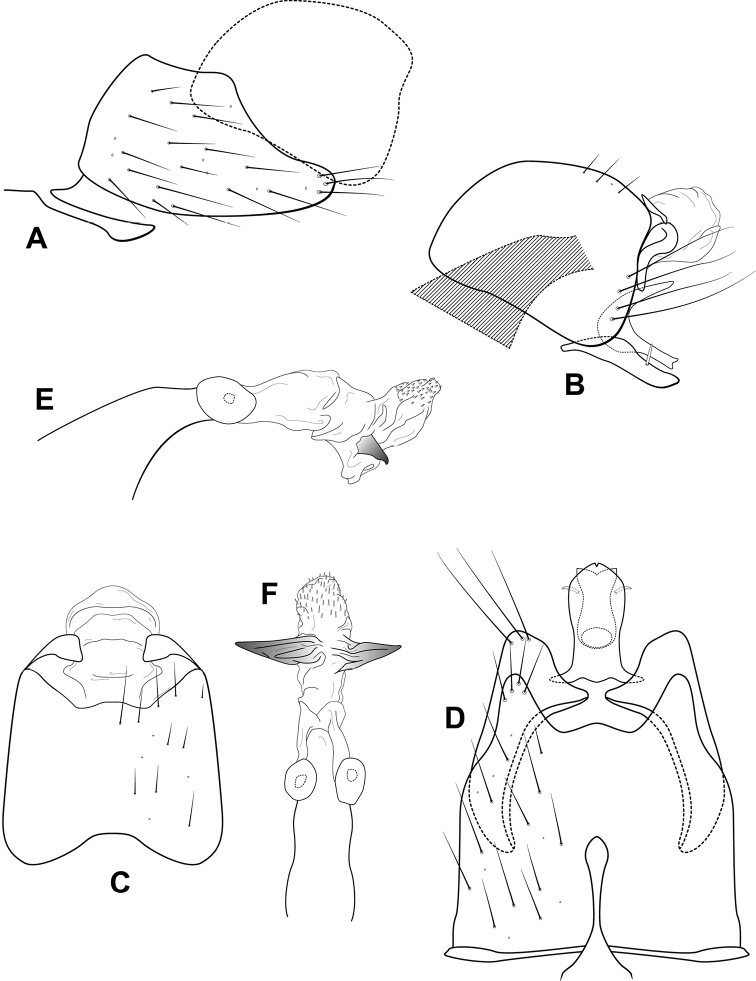
*Leucotrichiacortadera* sp. nov. Male genitalia: **A** segments VII–VIII and segment IX margin, lateral **B** segments IX–X, lateral (base of phallus crosshatched) **C** segments IX–X, dorsal **D** segments VII–IX, ventral **E** phallus, lateral **F** phallus, dorsal.

**Male.** Length of forewing 2.1 mm (*n* = 2). Wings unmodified. Head unmodified, with three ocelli; antennae unmodified. Tibial spur count 1, 3, 4. Color in alcohol brown. ***Genitalia*.** Abdominal sternum VII mesoventral process with enlarged apex (Fig. 5A, D). Sternum VIII with rounded posteroventral production in lateral view (Fig. 5A); in ventral view, posterior margin concave with broadly rounded mesal projection (Fig. 5D). Segment IX anterolateral margin convex, posterolateral margin straight with slight irregularity (Fig. 5B); dorsally, anterior margin concave, posterior margin concave with broadly rounded mesal projection (Fig. 5C). Tergum X with dorsal sclerite small, irregular; ventral sclerite with upper half rounded and bent posteriad; membranous apex suborbicular (Fig. 5B, C). Subgenital plate with dorsal arm simple, extending dorsad, apex acute (Fig. 5B); ventral arm simple, apex with emargination, in ventral view slightly restricted mesally, apex with broad emargination (Fig. 5B, D). Inferior appendage narrow basally, broadest submesally, with single dorsal subapical spine (Fig. 5B); ventrally entirely fused, broadest mesally, apex rounded with small pointed mesal emargination (Fig. 5D). Phallus tubular basally, constricted at midlength with median complex bearing spherical “windows”; apex membranous and bearing pair of large, acute sclerotized plates and numerous small apical spines on dorsal and lateral surface (Fig. 5E, F).

###### Distribution.

Panama.

###### Etymology.

The specific epithet is derived from *cortadera*, Spanish for “knife, cutting instrument”, referring to the shape of the large sclerotized plates found on the phallus apex.

##### ﻿ Leucotrichia cultrata

Taxon classificationAnimaliaTrichopteraHydroptilidae

Thomson & Armitage, 2021

00FA4B04-00A9-5D16-AFEB-75B1FB054561

Fig. 6

###### Diagnosis.

*Leucotrichiacultrata* is similar to *L.hispida* and *L.viridis*. The phallus of all three species shares a similar appearance with the basal loop of the median complex extended on basal supports and a ventral “bulge” to the membranous apex. The mesoventral process of sternum VII can be used to separate the three, being long and digitate in *L.cultrata*, bearing a tuft of prominent apical setae in *L.hispida*, and enlarged and apically blunt when viewed ventrally in *L.viridis*.

**Figure 6.**
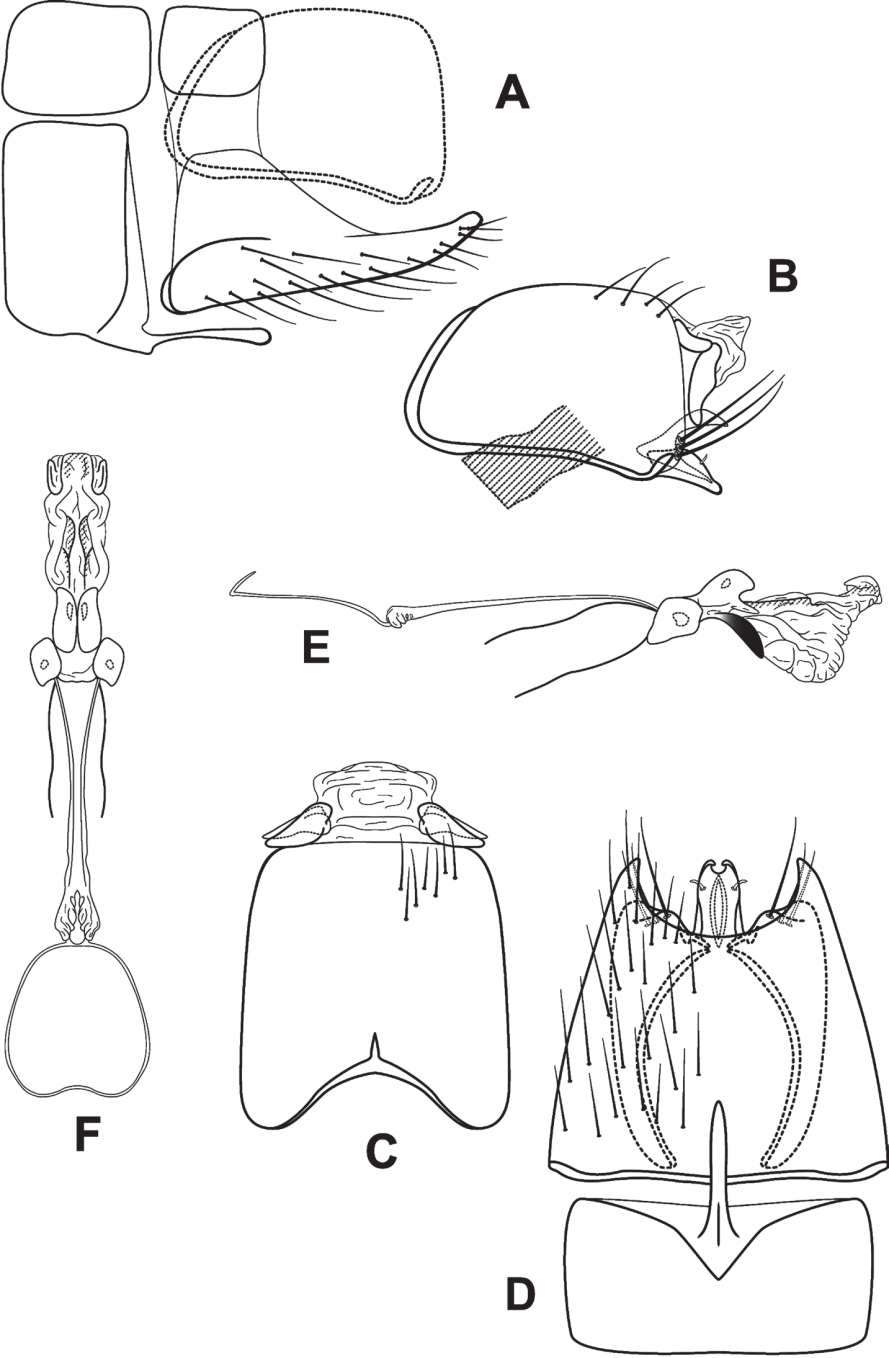
*Leucotrichiacultrata* Thomson & Armitage, 2021. Male genitalia: **A** segments VII–VIII and segment IX margin, lateral **B** segments IX–X, lateral (base of phallus crosshatched) **C** segments IX–X, dorsal **D** segments VII–IX, ventral **E** phallus, lateral **F** phallus, dorsal. Modified from Thomson and Armitage (2021).

###### Material examined.

**Panama: Bocas del Toro Province** • 2 males; Cuenca 093; Chiriqui Grande District; Quebrada Rambala, Rambala Jungle Lodge, 8.91627°N, 82.15469°W; 120 m a.s.l.; 9 Aug. 2014; E. Carlson, leg.; UV light trap; in alcohol; MUPADI • ibid., 5 males; 28 Mar. 2015 • ibid., 2 males; 31 Mar.–11 Apr. 2015; Malaise trap • ibid., 17 males; 12–15 Nov. 2017. **Chiriqui Province** • 1 male; Cuenca 102; Tierras Altas District; Mount Totumas Cloud Forest and Biological Reserve, Quebrada Norte; 8.873613°N, 82.690512°W; 1,709 m a.s.l.; 10–15 Jul. 2018; J. Dietrich, leg.; Malaise trap; in alcohol; MIUP • ibid., 2 males; 8–11 Nov. 2018; UMSP • ibid., 1 male; Cuenca 108; Dolega District, Río Majagua, Banquito de Palmira, Potrerillos; 8.68083°N, 82.53250°W; 840 m a.s.l.; 28 Feb.–14 Mar. 2019, Y. Aguirre, T. Ríos, leg.; Malaise trap (M001); in alcohol; MUPADI • ibid., 1 male; Quebrada Jaramillo, Finca Monterey; 8.76320°N, 82.41383°W; 1,214 m a.s.l.; 8–12 May 2018; B. Armitage, T. Arefina-Armitage, leg.; Malaise trap; in alcohol; MUPADI. **Panama Oeste Province** • 2 males; Cuenca 115; Altos de Campana National Park, Río Chileno, PSPSCB-PNAC-C115-2018-028; 8.71650°N, 80.00740°W; 497 m a.s.l.; 23–31 May 2018; T. Ríos, Y. Aguirre, leg.; Malaise trap; in alcohol; COZEM. **Veraguas Province** • 2 males; Cuenca 097; Santa Fe District; Santa Fe National Park; Río Calovébora, PSPSCB-PNSF-C097-2017-006; 8.55038°N, 81.16486°W; 461 m a.s.l.; 23–27 Apr. 2017, A. Cornejo, T. Ríos, E. Álvarez, C. Nieto, leg.; Malaise Trap; in alcohol; COZEM • ibid., 1 male; Cuenca 132; Río Mulaba, afl. 1er Brazo, PSPSCB-PNSF-C132-2017-008; 8.51706°N, 81.12140°W; 770 m a.s.l.; 19–23 Apr. 2017; T. Ríos, E. Álvarez, C. Nieto, leg.; Malaise trap; in alcohol; COZEM.

###### Distribution.

Panama.

##### ﻿ Leucotrichia extraordinaria

Taxon classificationAnimaliaTrichopteraHydroptilidae

Bueno-Soria, Santiago-Fragoso & Barba-Álvarez, 2001

436018A8-84B5-56C6-BF3F-5E25D504862B

Fig. 7

###### Diagnosis.

This species is similar to *L.dianeae* Thomson & Holzenthal, 2015 and *L.tapantia* Thomson & Holzenthal, 2015, two species originally described from Costa Rica that could potentially be collected in Panama (Table 4). In all three species, the posterolateral margin of sternum VIII is notably produced. Additionally, all three share a similar shape to the phallus apex, with bears a pair of apical lobes and lacks any spines or externally sclerotized structures. *Leucotrichiaextraordinaria* can be easily separated by the single peg-like apical seta on the posterolateral production of sternum VIII, a feature not shared with the other two species.

**Figure 7.**
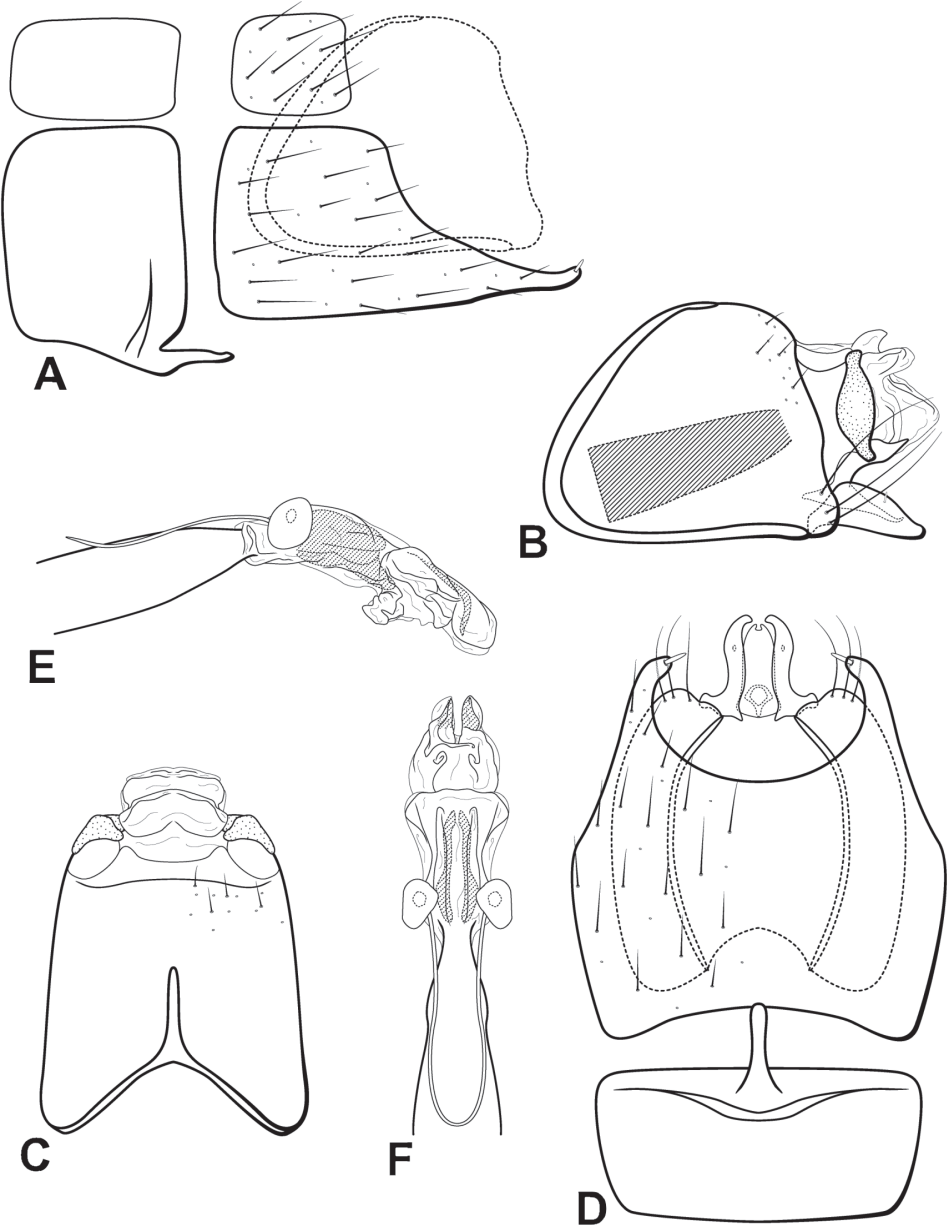
*Leucotrichiaextraordinaria* Bueno-Soria, Santiago-Fragoso & Barba-Álvarez, 2001. Male genitalia: **A** segments VII–VIII and segment IX lateral, lateral **B** segments IX–X, lateral (base of phallus crosshatched) **C** segments IX–X, dorsal, **D** segments VII–IX, ventral **E** phallus, lateral **F** phallus, dorsal. Modified from Thomson and Holzenthal (2015).

###### Material examined.

**Panama: Bocas del Toro Province** • 59 males; Cuenca 093; Chiriqui Grande District; Quebrada Rambala; Rambala Jungle Lodge; 8.91627°N, 82.15469°W; 120 m a.s.l.; 7–9 Oct. 2016; E. Carlson, leg.; Malaise trap; in alcohol; MUPADI • ibid., 18 males; 15–20 Nov. 2016; MUPADI • ibid., 13 males; 21–31 Dec. 2016; MUPADI; ibid., 6 males; 6–12 Feb. 2017; MUPADI • ibid., 18 males; 12–15 Jun. 2017; MUPADI • ibid. 3 males; 28–30 Jun. 2017; MUPADI. **Chiriqui Province** • 1 male; Cuenca 108; Boquete District; Quebrada Grande; Valle Escondido; 8.7797°N, 82.44016°W; 1,122 m a.s.l.; 29 Apr.–2 May 2018; B. Armitage, T. Arefina-Armitage, leg.; Malaise trap; in alcohol; MUPADI • ibid., 1 male; Quebrada Jaramillo, Finca Monterey; 8.7632°N, 82.41383°W; 1,214 m a.s.l.; 12–19 Aug. 2018; K. Collier, leg.; Malaise trap; in alcohol; MIUP • ibid., 2 males; 14–22 Oct. 2018 • ibid., 3 males; Dolega District, Río Majagua, Potrerillos, Banquito de Palmira; 8.68083°N, 82.532528°W; 840 m a.s.l.; 28 Feb–14 Mar. 2019; T. Ríos, Y. Aguirre, leg.; Malaise trap; in alcohol; UMSP • ibid., 3 males; Río Chirigagua, SSE Guayabal; 8.64102°N, 82.5578°W; 751 m a.s.l.; 19 Jun. 2015; C. Nieto, T. Abrego, E. Pérez, A. Tuñon, M. Molinar, A. Cornejo, leg.; UV light trap; in alcohol; COZEM. **Veraguas Province** • 2 males; Cuenca 097; Santa Fe District; Santa Fe National Park; afl. Río Calovébora; PSPSCB-NPSF-C-097-2017-005; 8.54318°N, 81.16398°W; 515 m a.s.l.; 19 Apr. 2017; A. Cornejó, T. Ríos, E. Álvarez, C. Nieto, leg.; UV light trap; in alcohol; COZEM • 1 male; Río Calovébora, PSPSCB-PNSF-C097-2017-006, 8.55038°N, 81.16486°W; 461 m a.s.l., 23–27 Apr. 2017; A. Cornejo, T. Ríos, E. Álvarez, C. Nieto, leg.; Malaise Trap; in alcohol; COZEM • ibid., 39 males; Cuenca 132, Río Mulaba, 2do Brazo, PSPSCB-NPSF-C-132-2017-007; 8.52577°N, 81.13045°W; 623 m a.s.l.; 19–23 Apr. 2017; Malaise trap; COZEM • ibid., 7 males; Río Piedra de Moler; PSPSCB-NPSF-C-097-2017-011; 8.55343°N, 81.17675°W; 395 m a.s.l.; 20 Apr. 2017; COZEM.

###### Distribution.

Mexico, Panama.

##### ﻿ Leucotrichia hispida

Taxon classificationAnimaliaTrichopteraHydroptilidae

Thomson & Holzenthal, 2015

70331CCB-8F30-5C21-8D30-C9E3D6E91ED4

Fig. 8

###### Diagnosis.

This species is similar to *L.botosaneanui*, *L.chiriquiensis*, and *L.viridis*, based on characteristics of the phallus, as discussed under *L.botosaneanui*. *Leucotrichiahispida* can be recognized using the tuft of setae on the posteroventral projection of sternum VIII and the lack of any external spines or sclerites on the apex of phallus.

**Figure 8.**
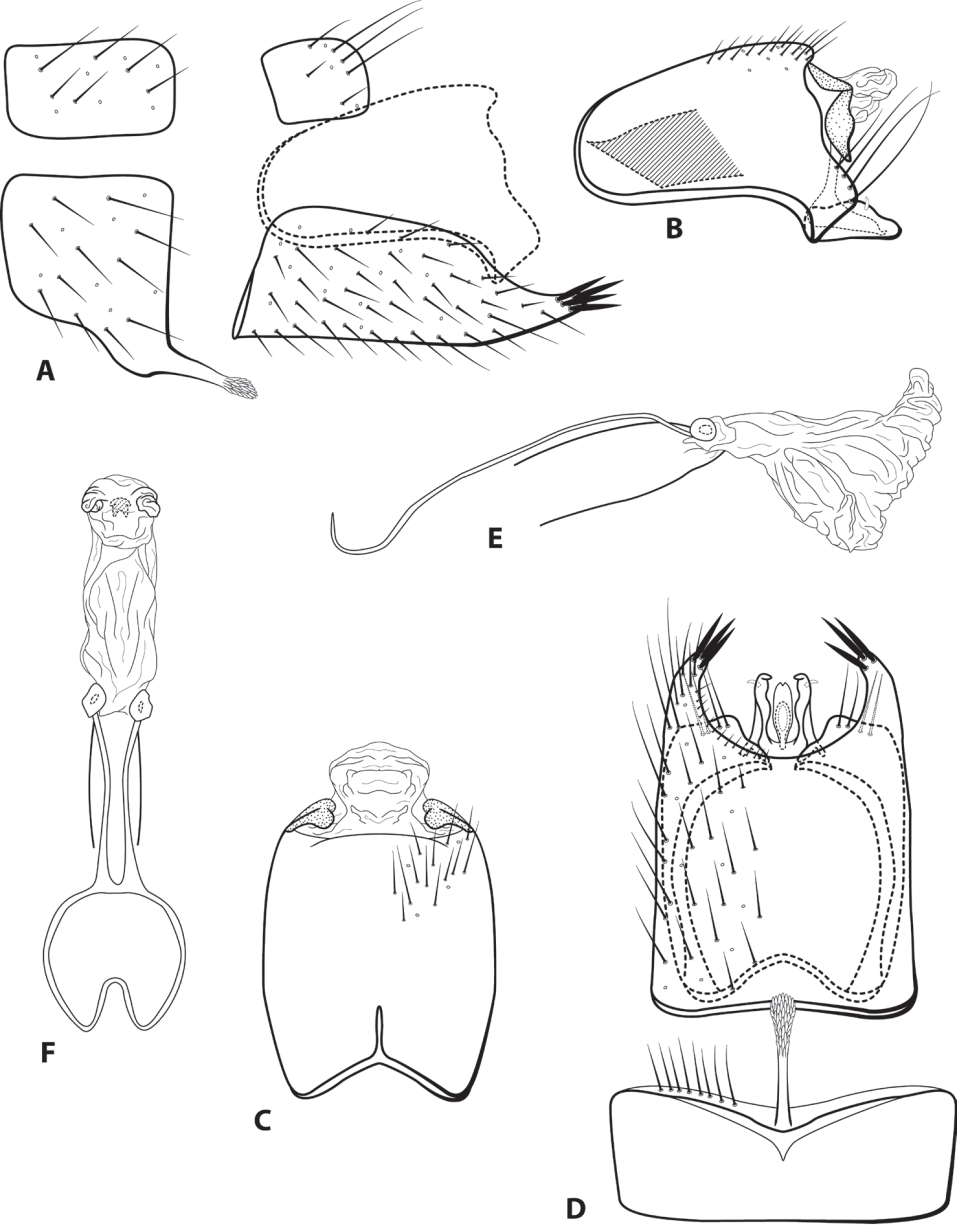
*Leucotrichiahispida* Thomson & Holzenthal, 2015. Male genitalia: **A** segments VII–VIII and segment IX margin, lateral **B** segments IX–X, lateral (base of phallus crosshatched) **C** segments IX–X, dorsal **D** segments VII–IX, ventral **E** phallus, lateral **F** phallus, dorsal. Modified from Thomson and Holzenthal (2015).

###### Material examined.

**Panama: Chiriqui Province** • 1 male; Cuenca 102; Renacimiento District; La Amistad International Park, Río Candela, Finca Felix, PSPSCB-PILA-C102-2017-021; 8.890557°N, 82.61201°W; 1,996 m a.s.l.; 25 Jan. 2015; C. Nieto, E. Pérez, A. Cornejo, leg.; UV light trap; in alcohol; COZEM • ibid., 1 male; Río Chiriqui Viejo, PSPSCB-PNVB-C108-2017-016; 8.87550°N, 82.55336°W; 2,117 m a.s.l.; 5–8 Jun. 2017; E. Álvarez, T. Ríos, E. Pérez, leg.; Malaise trap; COZEM • ibid., 2 males; afl. Río Chiriqui Viejo, PSPSCB-PILA-C102-2017-022; 8.90124°N, 82.61817°W; 2,354 m a.s.l.; 17–21 Jun. 2017; UMSP • ibid., 2 males; Cuenca 108; Quebrada del Guayabo, Volcan Baru National Park, PSPSCB-PNVB-C108-2017-018; 8.84939°N, 82.49349°W; 1,947 m a.s.l.; 5–8 Jun. 2017; E. Álvarez, E. Pérez, T. Ríos, leg.; Malaise trap; in alcohol; MUPADI.

###### Distribution.

Costa Rica, Panama.

##### ﻿ Leucotrichia holzenthali sp. nov.

Taxon classificationAnimaliaTrichopteraHydroptilidae

4D444D0A-DD44-5557-A999-F0D04C293441

http://zoobank.org/A5A3CC50-0E9D-490F-B042-EA3E3E4D7412

Fig. 9

###### Type locality.

**Panama: Veraguas Province**: Cuenca 097; Santa Fe District; Santa Fe National Park; Río Piedra de Moler; PSPSCB-PNSF-C097-2017-011; 8.55343°N, 81.17675°W; 395 m a.s.l.

###### Type material.

***Holotype***: male, **Panama: Veraguas Province**: Cuenca 097; Santa Fe District; Santa Fe National Park; Río Piedra de Moler; PSPSCB-PNSF-C097-2017-011; 8.55343°N, 81.17675°W; 395 m a.s.l.; 20 Apr. 2017; A. Cornejo, T. Ríos, E. Álvarez, C. Nieto, leg.; UV light trap; in alcohol; MIUP-002-T-2021. ***Paratype***: same data as for holotype; 1 male; UMSP.

###### Diagnosis.

*Leucotrichiaholzenthali* sp. nov., is similar to *L.dinamica* Bueno-Soria, 2010, a species currently known only from Mexico. Both species bear a pair of large scissor-like sclerites on the apex of the phallus. *Leucotrichiaholzenthali* can be distinguished by the additional pair of ventral sclerites on the phallus apex and the peg-like setae on abdominal sternum VIII, both characteristics that are absent on *L.dinamica*.

###### Description.

**Figure 9.**
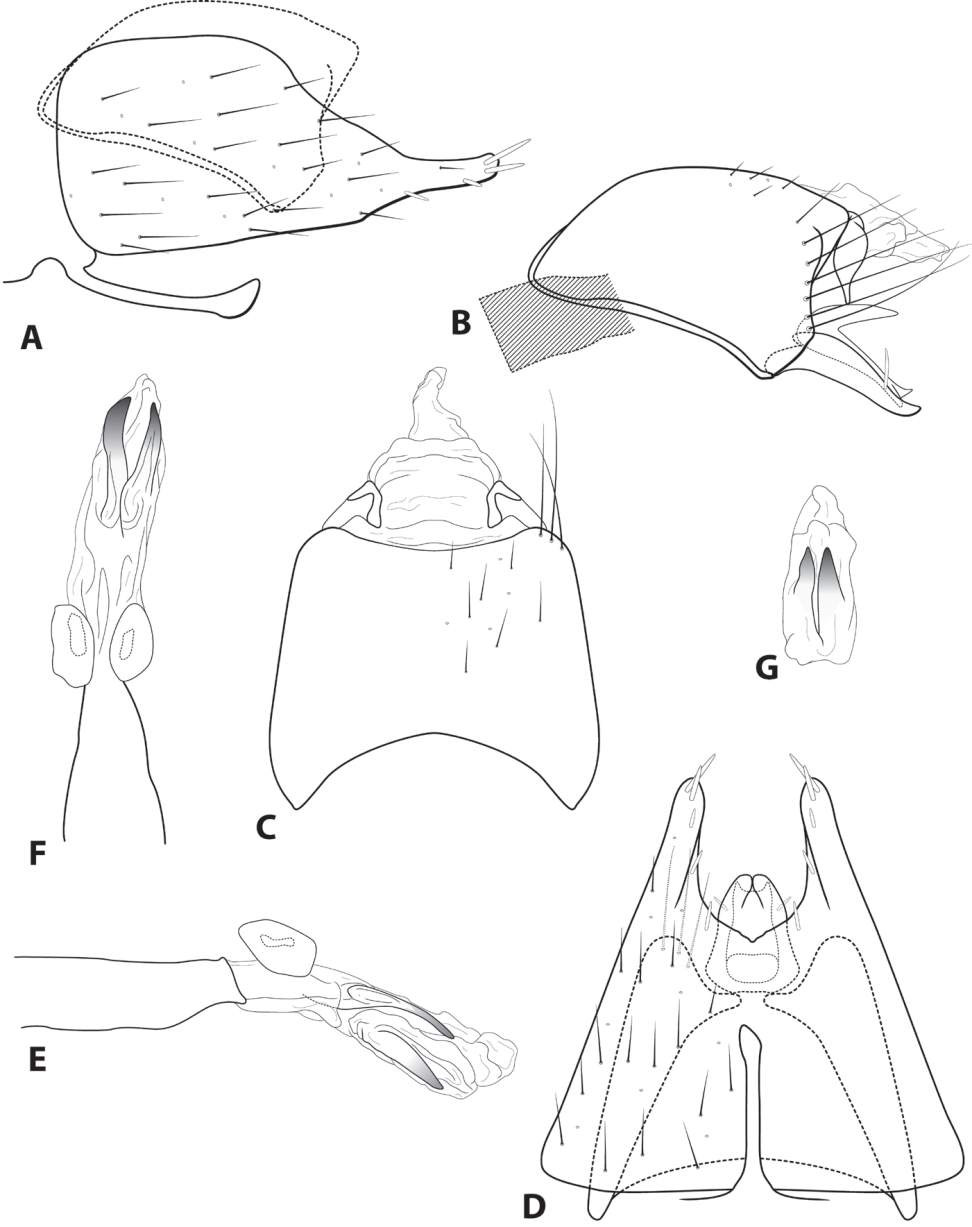
*Leucotrichiaholzenthali* sp. nov. Male genitalia: **A** segments VII–VIII and segment IX margin, lateral **B** segments IX–X, lateral (base of phallus crosshatched) **C** segments IX–X, dorsal **D** segments VII–IX, ventral **E** phallus, lateral **F** phallus, dorsal **G** phallus apex, ventral.

**Male.** Length of forewing 1.7 mm (*n* = 2). Wings unmodified. Head unmodified, with three ocelli; antennae unmodified. Tibial spur count 1, 3, 4. Color in alcohol brown. ***Genitalia*.** Abdominal sternum VII mesoventral process with enlarged apex (Fig. 9A, D). Sternum VIII with posteroventral production bearing prominent peg-like setae (Fig. 9A), in ventral view posterior margin concave (Fig. 9D). Segment IX anterolateral margin convex, posterolateral margin irregular (Fig. 9B); dorsally, anterior margin concave, posterior margin broadly concave (Fig. 9C). Tergum X with dorsal sclerite not apparent; ventral sclerite broadest mesally with slender ventral apex; membranous apex subtriangular in dorsal view (Fig. 9B, C). Subgenital plate with dorsal arm simple, extending posteriad, apex acute (Fig. 9B); ventral arm simple, apex with emargination, in ventral view with truncate base and rounded apical emargination (Fig. 9B, D). Inferior appendage broadest basally, with pointed basal emargination, prominent dorsal subapical seta, apex acute (Fig. 9B); ventrally broadly fused, broadest basally, apex rounded (Fig. 9D). Phallus tubular basally, constricted at midlength with median complex bearing spherical “windows”; apex membranous, bearing pair of large scissor-like apical sclerites dorsally and pair of large acute sclerites ventrally (Fig. 9E, F).

###### Distribution.

Panama.

###### Etymology.

Named in honor of Dr. Ralph W. Holzenthal, for a long and robust career in caddisfly taxonomy and systematics. Dr. Holzenthal has been a friend and colleague to each of the authors, and an invaluable mentor in particular to the first author.

##### ﻿ Leucotrichia luma sp. nov.

Taxon classificationAnimaliaTrichopteraHydroptilidae

3034911A-6166-5EDC-9A09-618087B3C859

http://zoobank.org/C11BC765-4839-4131-8D82-04EFB0045C1A

Fig. 10

###### Type locality.

**Panama: Panama Oeste Province**: Cuenca 115; Altos de Campana National Park, Río Chileno, PSPSCB-PNAC-C115-2018-028; 8.716502°N, 80.00740°W; 497 m a.s.l.

###### Type material.

***Holotype***: male, **Panama: Panama Oeste Province** • Cuenca 115; Altos de Campana National Park, Río Chileno, PSPSCB-PNAC-C115-2018-028; 8.716502°N, 80.00740°W; 497 m a.s.l.; 27–31 May 2018, T. Ríos, Y. Aguirre, leg.; Malaise trap; in alcohol; COZEM; MIUP-003-T-2021. ***Paratypes***: ibid., 4 males; COZEM and UMSP • ibid., 12 males; Cuenca 138; Río Sajalice, PSPSCB-PNAC-C138-2018-030; 8.67625°N, 79.89748°W; 194 m a.s.l.; 27–31 May 2018; Malaise trap; in alcohol; COZEM and MUPADI.

###### Diagnosis.

*Leucotrichialuma* is most similar to *L.inflaticornis*, a species currently known only from Trinidad. Certain characteristics found on the genitalia of these species make them very similar, such as the symmetrical rows of spines on the phallus apex, a unique arrangement within the genus. Key differences, however, make it possible to separate the two. Inflated antennal segments, a key feature of *L.inflaticornis*, were not observed in any of the specimens collected in Panama and identified as *L.luma*. Additionally, *L.luma* specimens all present three pairs of spines on the phallus apex, while the original description of *L.inflaticornis* states that there should be four. The first author has observed the holotype specimen of *L.inflaticornis* and found that the abdomen, including the phallus, was missing and key features of the genitalia cannot be confirmed. Since specimens cannot be compared to the *L.inflaticornis* holotype, we compare these specimens to the original description and illustration and offer this new species description for the specimens from Panama.

###### Description.

**Figure 10.**
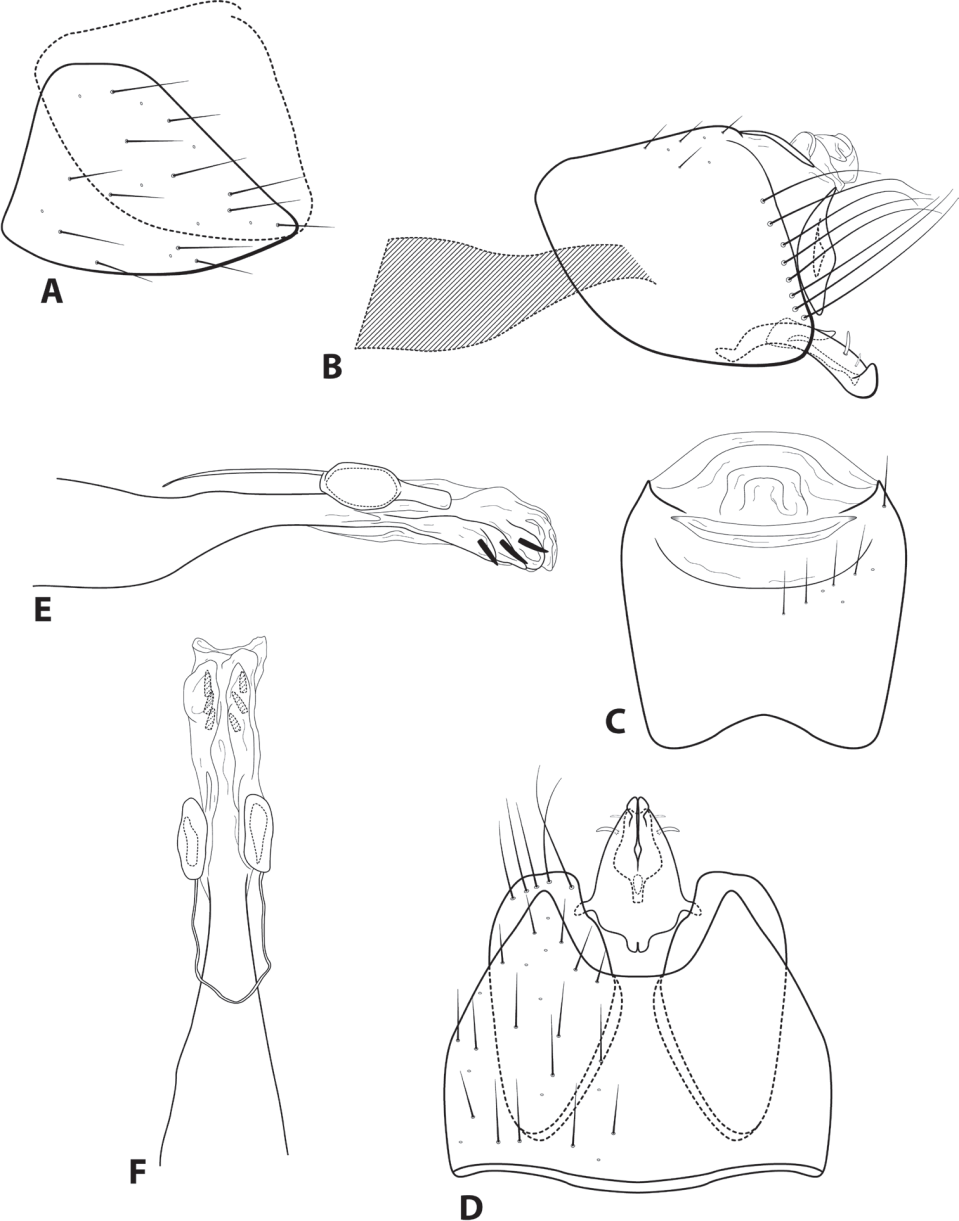
*Leucotrichialuma* sp. nov. Male genitalia: **A** segments VII–VIII and segment IX margin, lateral **B** segments IX–X, lateral (base of phallus crosshatched) **C** segments IX–X, dorsal **D** segments VII–IX, ventral **E** phallus, lateral **F** phallus, dorsal.

**Male.** Length of forewing 1.8–2.0 mm (*n* = 17). Wings unmodified. Head unmodified, with 3 ocelli; antennae unmodified. Tibial spur count 1, 3, 4. Color in alcohol brown. ***Genitalia*.** Abdominal sternum VII without apparent mesoventral process. Sternum VIII with acute posteroventral production, in ventral view posterior margin concave (Fig. 10A, D). Segment IX anterolateral margin convex, posterolateral margin straight (Fig. 10B); dorsally, anterior margin concave, posterior margin concave (Fig. 10C). Tergum X with dorsal sclerite simple, slender; ventral sclerite semi-elliptical with rounded emargination mesally on posterior margin; membranous apex small, suborbicular (Fig. 10B, C). Subgenital plate with dorsal arm digitate, approximately half the length of ventral arm (Fig. 10B); ventral arm slender, apex truncate, with irregular ventral margin, in ventral view broadest mesally with rounded apical emargination (Fig. 10B, D). Inferior appendage with base extending anteriorad, with two dorsal subapical spines, apex curved dorsad (Fig. 10B); in ventral view broadly fused, with digitate basal projections (Fig. 10D). Phallus tubular basally, constricted at midlength with median complex bearing basal loop and pair of spherical “windows”; apex membranous and bearing 3 sets of symmetrically arranged stout, dark spines (Fig. 10E, F).

###### Distribution.

Panama.

###### Etymology.

The specific epithet is derived from *luma*, Latin for “thorn”, referring to the spines found on the phallus apex.

##### ﻿ Leucotrichia melleopicta

Taxon classificationAnimaliaTrichopteraHydroptilidae

Mosely, 1934

4AFA0BB6-0AA1-54A1-A9F3-2C6877EBE447

Fig. 11

###### Diagnosis.

**Figure 11.**
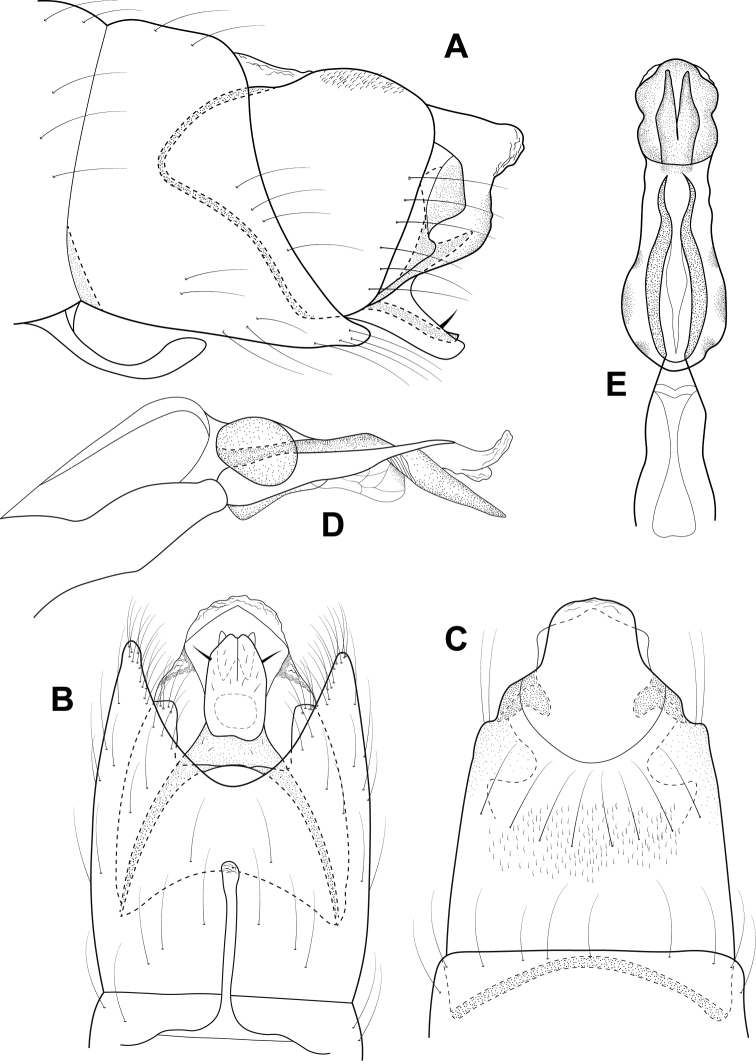
*Leucotrichiamelleopicta* Mosely, 1934. Male genitalia: **A** segments VII–X, lateral **B** segments VIII–X, ventral **C** segments IX–X, dorsal **D** phallus, lateral **E** phallus dorsal. Modified from Harris and Armitage (2019).

*Leucotrichiamelleopicta* is most similar to *L.mutica*, also recorded from Panama. These species possess an inferior appendage with a similar shape, and the dorsal sclerite of the phallus of both bears a dorsal sclerite with a distinct apical emargination. *Leucotrichiamelleopicta* can be distinguished by the elongate basal sclerite of the phallus and the poorly developed basal loop (Fig. 11E), and by the enlarged apex of the mesoventral process on segment VII (Fig. 11A).

###### Material examined.

**Panama: Bocas del Toro Province** • 6 males; Cuenca 093; Chiriqui Grande District; Quebrada Rambala; Rambala Jungle Lodge; 8.91627°N, 82.15469°W; 120 m a.s.l.; 28 Mar. 2015; B. Armitage, T. Arefina- Armitage, leg.; UV light trap; in alcohol; MUPADI • ibid., 68 males; 31 Mar.–11 Apr. 2015; E. Carlson, leg.; Malaise trap • ibid., 38 males; 7–9 Oct. 2016 • ibid., 36 males; 15–20 Nov. 2016 • ibid., 66 males; 21–31 Dec. 2016 • ibid., 55 males; 6–12 Feb. 2017 • ibid., 32 males; 12–15 Jun. 2017 • ibid., 17 males; 28–20 Jun. 2017. **Comarca Ngäbe Buglé** • 1 male; Cuenca 093; Palo Seco Forest Preserve; Quebrada Martinez; Alto de Valle, detrás de las caseta de MiAmbiente; 8.79484°N, 82.19047°W; 480 m a.s.l.; 5–19 May 2019; T. Ríos, Y. Aguirre, leg.; Malaise trap; in alcohol; MUPADI • ibid., 4 males; 24 May–6 Jun. 2019 • ibid., 5 males; 22 Sep.–11 Oct. 2019 • ibid., 2 males; Willie Mazu, 8.79361°N, 82.19391°W, 538 m a.s.l.; 13–27 Sep. 2019 • ibid., 1 male; 11–30 Oct. 2019. **Chiriqui Province** • 27 males; Cuenca 108, Boquete District; Quebrada Jaramillo; Finca Monterey; 8.7632°N, 82.41383°W; 1,214 m a.s.l.; 19–25 Apr. 2018, K. Collier, leg.; Malaise trap; in alcohol; MUPADI • ibid., 41 males; 8–12 May 2018 • ibid., 54 males; 16–20 Jun. 2018 • ibid., 5 males; 12–19 Aug. 2018 • ibid., 52 males; 14–22 Oct. 2018 • ibid., 6 males; 15–22 Nov. 2018 • ibid., 48 males; Dolega District, Río Majagua, Potrerillos, Banquito de Palmira; 8.68083°N, 82.532528°W; 840 m a.s.l.; 28 Feb–14 Mar. 2019; T. Ríos, Y. Aguirre, leg.; Malaise trap; in alcohol; UMSP. **Veraguas Province** • 2 males; Cuenca 097, Santa Fe District, Santa Fe National Park, afl. Río Calovébora; PSPSCB-NPSF-C-097-2017-005; 8.54318°N, 81.16398°W; 515 m a.s.l.; 19–23 Apr. 2017; A. Cornejo, T. Ríos, E. Álvarez, C. Nieto, leg.; Malaise trap; in alcohol; COZEM • ibid., 1 male; Río Llanito, PSPSCB-NPSF-C-097-2017-012; 8.56553°N, 81.18817°W; 340 m a.s.l.; 21 Apr. 2017; UV light trap; COZEM • ibid., 1 male; Río Pedra Moler; PSPSCB-NPSF-C-097-2017-011; 8.55343°N, 81.17675°W; 395 m a.s.l.; 20 Apr. 2017 • ibid., 104 males; Cuenca 132, Río Mulaba, 2do Brazo, PSPSCB-NPSF-C-097-2017-007; 8.52577°N, 81.13045°W; 623 m a.s.l.; 19–23 Apr. 2017; Malaise trap; COZEM • ibid., 41 males; Río Mulaba, afl. 1er Brazo; PSPSCB-NPSF-C-097-2017-008; 8.51706°N, 81.1214°W; 770 m a.s.l.; 19–23 Apr. 2017; COZEM.

###### Distribution.

Mexico, Panama, Venezuela.

###### Remarks.

In the paper Harris and Armitage (2019), *Leucotrichamelleopicta* was redrawn to compare with *L.mutica* as both species were common throughout Panama. In Thomson and Holzenthal (2015) the drawings were prepared from material collected in Mexico and compared to that of Venezuela. There were no comparisons with specimens from Central America. The drawings of genitalic features in the two publications are very similar. However, in the material from Mexico there was a posterior cleft in the ventral arm of the subgenital plate, which was not observed in material from Panama. This is a small character to observe and it could be present but not seen with the arm slightly turned, or it could be absent. The phallus drawings are similar, but in Harris and Armitage the subapical rods are separate, while in Thomson & Holzenthal they are fused. However, the rods are not fixed in position and there is some lateral movement. This is based on the large number of examined specimens from Panama. Likewise the phallic apical rods are fused basally in Harris and Armitage, while they are separate, but closely aligned in Thomson and Holzenthal. Both arrangements of the phallic rods were observed in the material from Panama.

##### ﻿ Leucotrichia mutica

Taxon classificationAnimaliaTrichopteraHydroptilidae

Flint, 1991

DB6C7A0C-7B2F-5D73-9547-ED458BE8D007

Fig. 12

###### Diagnosis.

**Figure 12.**
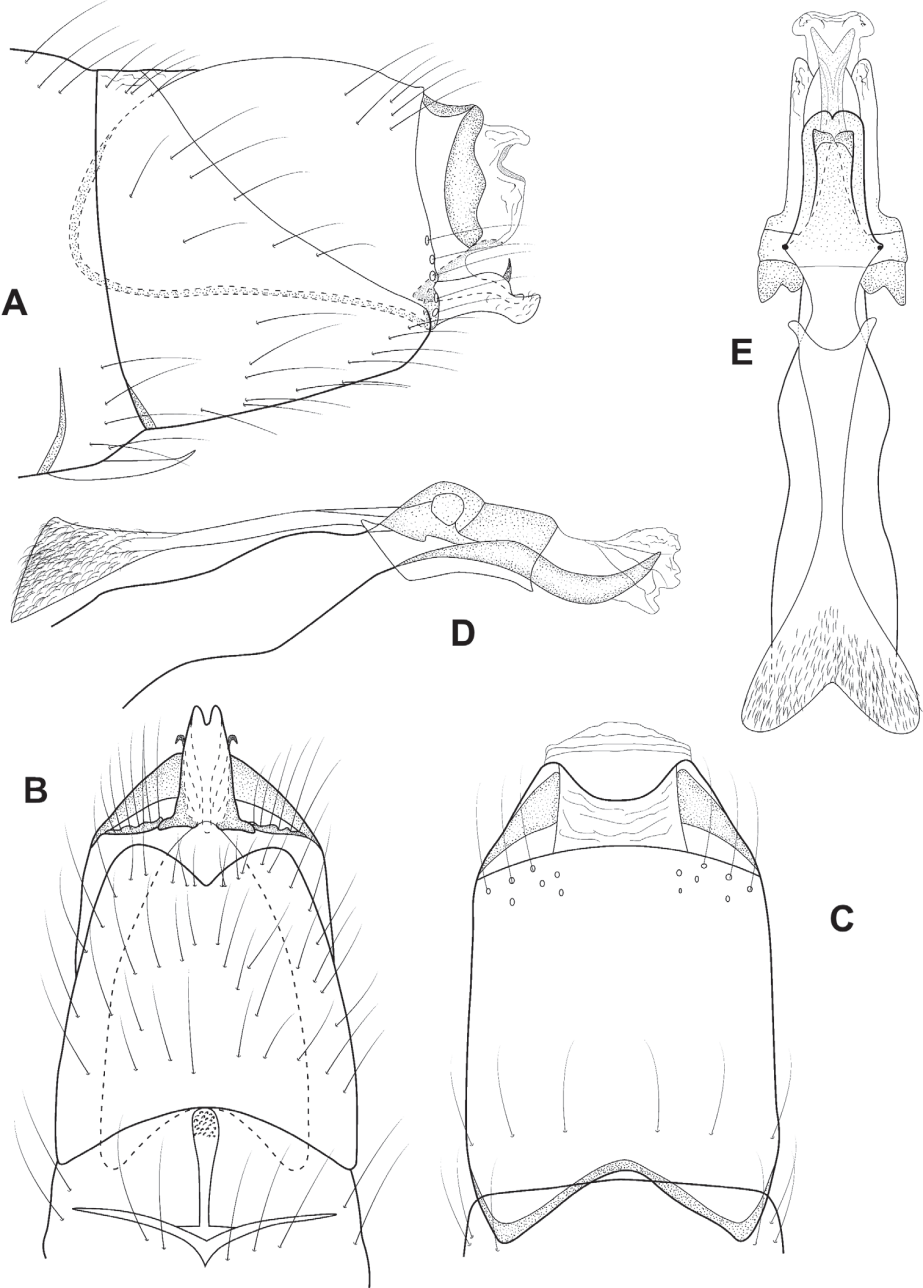
*Leucotrichiamutica* Flint, 1991. Male genitalia: **A** segments VII–X, lateral **B** segments VII–IX, ventral **C** segments IX–X, dorsal **D** phallus, lateral **E** phallus, dorsal. Modified from Harris and Armitage (2019).

This species is similar to *L.melleopicta*; both species display ranges that include Panama. As discussed under *L.melleopicta*, these species share similarities in the dorsal sclerite of the phallus apex, although this sclerite is much smaller than that seen in *L.melleopicta* (Fig. 12E), and the general shape of the inferior appendage. *Leucotrichiamutica* can be recognized separately from *L.melleopicta* by the tapering mesoventral process on segment VII (Fig. 12A), and by the prominent basal loop of the phallus (Fig. 12D, E).

###### Material examined.

**Panama: Bocas del Toro Province** • 17 males; Cuenca 093; Chiriqui Grande District; Quebrada Rambala; Rambala Jungle Lodge; 8.91627°N, 82.15469°W; 120 m a.s.l.; 7–9 Oct. 2016; E. Carlson, leg.; Malaise trap; in alcohol; MUPADI • ibid., 10 males; 15–20 Nov. 2016 • ibid., 6 males; 21–31 Dec. 2016 • ibid., 6 males; 6–12 Feb. 2017 • ibid., 14 males; 12–15 Jun. 2017 • ibid., 47 males; 28–30 Jun. 2017. **Comarca Ngäbe Buglé** • 1 male; Quebrada Martinez, Bosque Protector Palo Seco, Alto de Valle; detrás de las caseta de MiAmbiente, M0002; 8.79424°N, 82.1904724°W; 480 m a.s.l.; 24 May–6 Jun. 2018; Y. Aguirre, T. Ríos, leg.; Malaise trap; in alcohol; MUPADI. **Chiriqui Province** • 1 male; Cuenca 108; Boquete District; Quebrada Grande; Valle Escondido; 8.7797°N, 82.44016°W; 1,122 m a.s.l.; 11 Mar. 2018; T. Arefina-Armitage, leg.; UV light trap • 6 males; 29 Apr.–2 May 2018; Malaise trap • ibid., 1 male; 21 May 2018; UV light trap • ibid., 1 male; 8.783645°N, 82.444287°W; 1,147 m a.s.l.; 27–30 May 2018; Malaise trap • ibid., 1 male; 17–20 Jun. 2018 • 6 males; 23 Jul. 2018; UV light trap • ibid., 2 males; 10 Nov. 2018. **Veraguas Province** • 1 male; Cuenca 097; Santa Fe District; Santa Fe National Park; afl. Río Calovébora; PSPSCB-NPSF-C-097-2017-005; 8.54318°N, 81.16398°W; 515 m a.s.l.; 19–23 Apr. 2017; Malaise trap; in alcohol; COZEM.

###### Distribution.

Colombia, Panama.

###### Remarks.

*Leucotrichiamutica* in Thomson and Holzenthal (2015) was drawn from the holotype of the species from Colombia. This specimen had the lower half of the phallus missing from the medial ring-like structure downward, leaving only the apical portion. The species was fairly common in Panama, and we were able to identify it from the apical portion of the phallus, which in *Leucotrichia* is typically diagnostic, as well as the other genitalic features. Based on the fact that Thomson and Holzenthal’s original description was based on this single specimen from Colombia, we redrew it in Harris and Armitage (2019) to better reflect variation in the species and to include the basal portion of the phallus. The latter can be very useful in the diagnosis of the species.

##### ﻿ Leucotrichia rhomba

Taxon classificationAnimaliaTrichopteraHydroptilidae

Thomson & Holzenthal, 2015

56DF0027-0A45-5E3A-86B1-666B30D04600

Fig. 13

###### Diagnosis.

**Figure 13.**
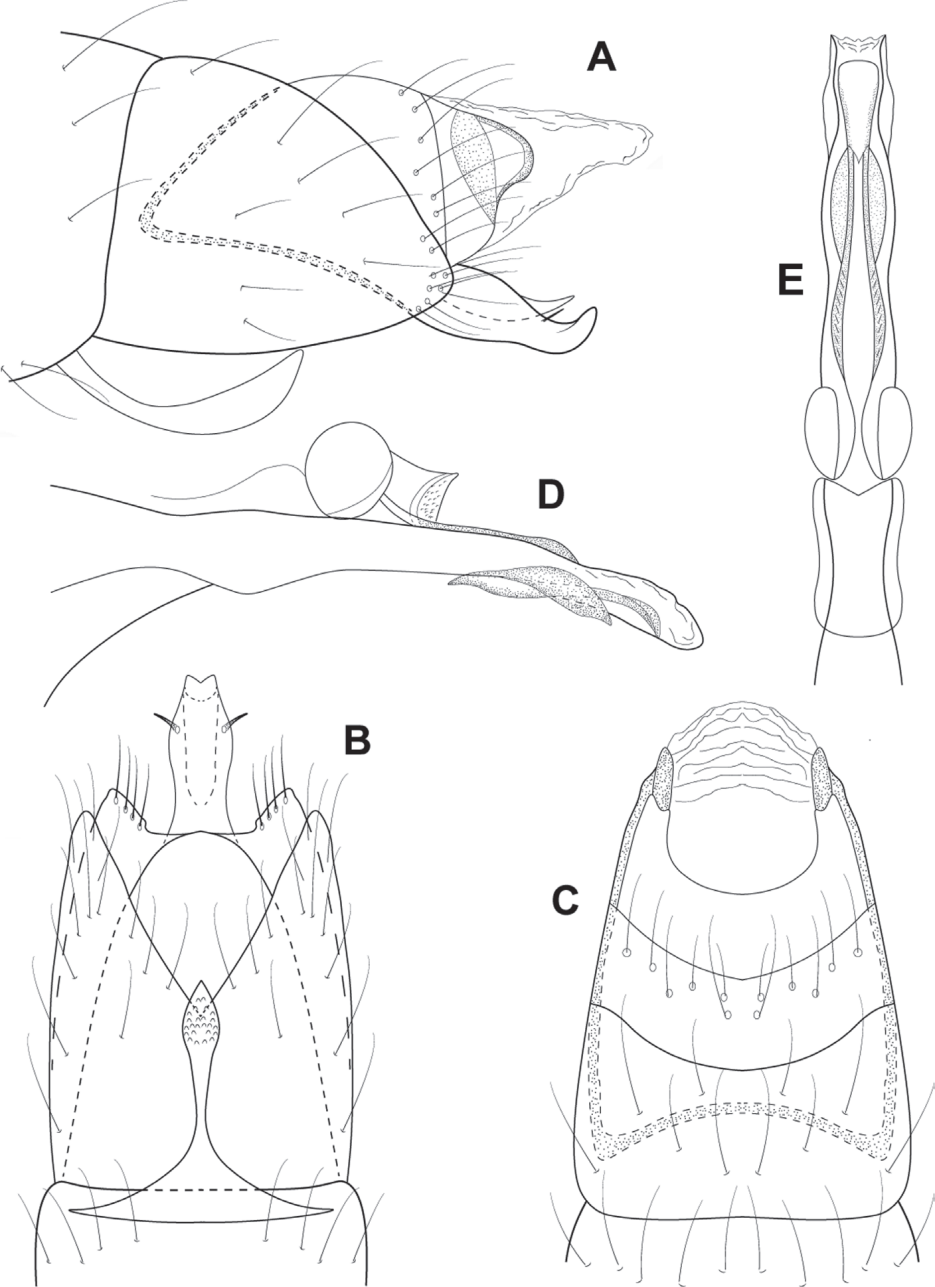
*Leucotrichiarhomba* Thomson & Holzenthal, 2015. Male genitalia: **A** segments VII–X, lateral **B** segments VII–IX, ventral **C** segments VII–X, dorsal **D** phallus, lateral **E** phallus, dorsal. Modified from Harris and Armitage (2019).

*Leucotrichiarhomba* is similar to *L.brochophora* Flint, 1991 and *L.padera* Flint, 1991, two species recorded from Colombia, but not currently known from Panama. All three species share a similar appearance of the phallus apex, with no spines and either few or no externally sclerotized structures. The anterolateral margin of segment IX is also produced in all three species. *Leucotrichiarhomba* can be recognized by the elongate mesoventral process of segment VII, which is enlarged, rhomboid, and rugose in ventral view (Fig. 13A, B), and by the obovoid dorsal sclerite of the phallus apex (Fig. 13E).

###### Material examined.

**Panama: Bocas del Toro Province** • 6 males; Cuenca 093; Chiriqui Grande District; Quebrada Rambala; Rambala Jungle Lodge; 8.91627°N, 82.15469°W; 120 m a.s.l.; 12–15 Jun. 2017; E. Carlson, leg.; Malaise trap; in alcohol; MUPADI • ibid., 1 male; 28–30 Jun. 2017 • ibid., 2 males; 15–20 Nov. 2016. **Chiriqui Province** • 5 males; Cuenca 108; Boquete District; Quebrada Jaramillo; Finca Monterey; 8.7632°N, 82.41383°W; 1,214 m a.s.l.; 16–20 Jun. 2017; K. Collier, leg.; Malaise trap • ibid., 1 male; 14­–22 Oct. 2018. • ibid., 5 males; Dolega District, Río Majagua, Potrerillos, Banquito de Palmira; 8.68083°N, 82.532528°W; 840 m a.s.l.; 28 Feb.–14 Mar. 2019; T. Ríos, Y. Aguirre, leg.; MUPADI. **Veraguas Province** • 14 males; Cuenca 132; Santa Fe District, Santa Fe National Park, Río Mulaba, 2do Brazo, PSPSCB-NPSF-C-097-2017-007; 8.52577°N, 81.13045°W; 623 m a.s.l.; 19–23 Apr. 2017; A. Cornejo, T. Ríos, E. Álvarez, C. Nieto, leg.; Malaise trap; in alcohol; COZEM.

###### Distribution.

Costa Rica, Panama.

###### Remarks.

The drawings of *Leucotrichiarhomba* in Harris and Armitage (2019) emphasize the apical phallic sclerite which is somewhat oval in shape. In Thomson and Holzenthal (2015) this sclerite is not emphasized, rather they use the basal sclerites. In Harris and Armitage (2019), these basal sclerites are indicated, but secondarily to the apical sclerite. In examining material from Panama, the apical sclerite proved to be the best character for identifying the species. The other genitalic features are similarly drawn in these two papers. The minor difference in figures could be attributed to artistic interpretation of taxonomic features.

##### ﻿ Leucotrichia ruiteri sp. nov.

Taxon classificationAnimaliaTrichopteraHydroptilidae

0AE63BE7-2735-5777-9552-39625A0FE4E0

http://zoobank.org/F7961E34-1751-4095-A5D1-27D5E29B698D

Figs 2A , 14

###### Type locality.

**Panama: Chiriqui Province**: Cuenca 108; Boquete District; Quebrada Jaramillo, Finca Monterey; 8.7632°N, 82.41383°W; 1,214 m a.s.l.

###### Type material.

***Holotype***: male, **Panama: Chiriqui Province**: Cuenca 108; Boquete District; Quebrada Jaramillo, Finca Monterey; 8.7632°N, 82.41383°W; 1,214 m a.s.l.; 8–12 Jun. 2018, K. Collier, leg.; Malaise trap; in alcohol; MIUP-004-T-2021. ***Paratype***: same data as for holotype; 1 male; UMSP.

###### Other material examined.

**Panama: Chiriqui Province** • 1 male; Cuenca 102, Renacimiento District; La Amistad International Park, Río Candela, Finca Felix, PSPSCB-PILA-C102-2017-021; 8.90614°N, 82.72882°W; 1,799 m a.s.l., 1–5 Sep. 2017; E. Álvarez, T. Ríos, E. Pérez, leg.; Malaise trap; in alcohol; COZEM. **Veraguas Province** • 1 male; Cuenca 132, Santa Fe National Park, Río Mulaba, 2do Brazo, PSPSCB-NPSF-C-097-2017-007; 8.52577°N, 81.13045°W; 623 m a.s.l.; 19–23 Apr. 2017; A. Cornejo, T. Ríos, E. Álvarez, C. Nieto, leg.; Malaise trap; in alcohol; MUPADI.

###### Diagnosis.

*Leucotrichiaruiteri* sp. nov. is most similar to *L.hispida*, as both species bear prominent setae on the posteroventral production of sternum VIII, a bilobed phallus apex, and a similar shaped inferior appendage. *Leucotrichiaruiteri* can be separated by the single, elongate seta on sternum VIII compared to the cluster of setae present on *L.hispida*. Additionally, the forewings of *L.ruiteri* are modified with a large setae-filled pocket, while those of *L.hispida* are unmodified.

###### Description.

**Figure 14.**
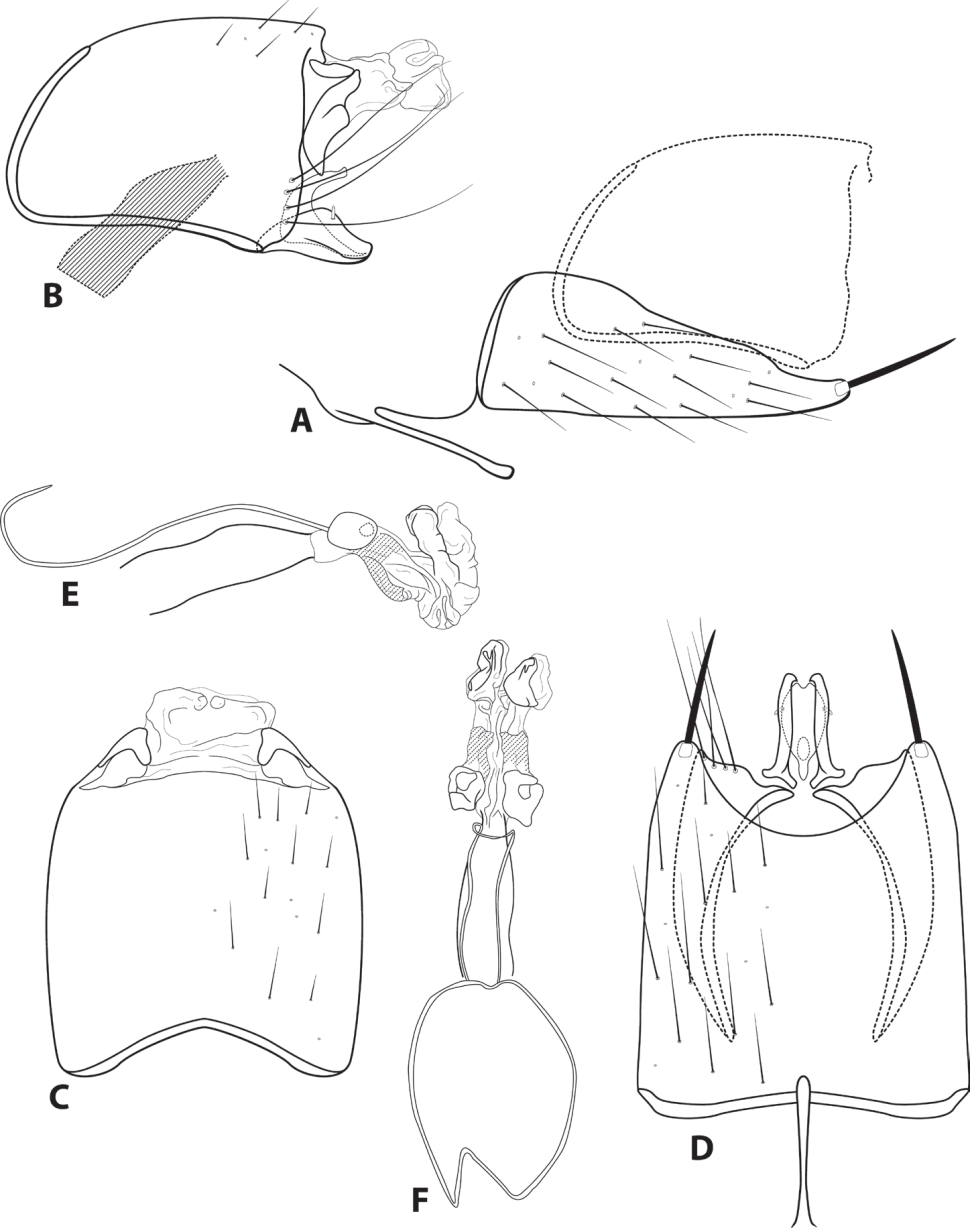
*Leucotrichiaruiteri* sp. nov. Male genitalia: **A** segments VII–VIII and segment IX margin, lateral **B** segments IX–X, lateral (base of phallus crosshatched) **C** segments IX–X, dorsal **D** segments VII–IX, ventral **E** phallus, lateral **F** phallus, dorsal.

**Male.** Length of forewing 2.0–2.1 mm (*n* = 4). Forewing with large pocket filled with scales (Fig. 2A); hindwing unmodified. Head unmodified, with three ocelli; antennae unmodified. Tibial spur count 1, 3, 4. Color in alcohol brown. ***Genitalia*.** Abdominal sternum VII slender, elongate (Fig. 14A, D). Sternum VIII with posteroventral production bearing prominent apical seta (Fig. 14A); in ventral view posterior margin concave (Fig. 14D). Segment IX anterolateral margin convex, posterolateral margin straight with slight irregularity (Fig. 14B); dorsally, anterior margin concave, posterior margin broadly convex (Fig. 14C). Tergum X with dorsal sclerite with irregular dorsal margin; ventral sclerite semi-elliptic with knoblike projection mesally on posterior margin; membranous apex subquadrate (Fig. 14B, C). Subgenital plate with dorsal arm simple, with slight preapical emargination on dorsal margin, apex truncate (Fig. 14B); ventral arm narrowing apically, ventral margin slightly sinuate, in ventral view subovate with narrow basal projection and small rounded apical emargination (Fig. 14B, D). Inferior appendage broadest mesally, with small dorsal subapical peg-like seta, apex rounded (Fig. 14B); ventrally slender, with digitate basal projections, apex slightly hooked on inner margin (Fig. 14D). Phallus tubular basally, constricted at midlength with typical median complex bearing basal loop and pair of spherical “windows”; apex membranous with internal sclerotized structures and two apical lobes extending dorsad (Fig. 14E, F).

###### Distribution.

Panama.

###### Etymology.

This species is named in honor and memory of Dave Ruiter, a passionate and enthusiastic caddisfly researcher and good friend, who recently passed away.

##### ﻿ Leucotrichia viridis

Taxon classificationAnimaliaTrichopteraHydroptilidae

Flint, 1967

CABCDD3C-EF21-54EE-B73F-B810E2F06FA9

Fig. 15

###### Diagnosis.

Due to the similar overall appearance of the phallus, *L.viridis* is most similar to *L.botosaneanui*, *L.hispida*, and *L.chiriquiensis*, as discussed under *L.botosaneanui*. *Leucotrichiaviridis* has two dorsal spines on the inferior appendage, while the other species each bear only a single spine.

**Figure 15.**
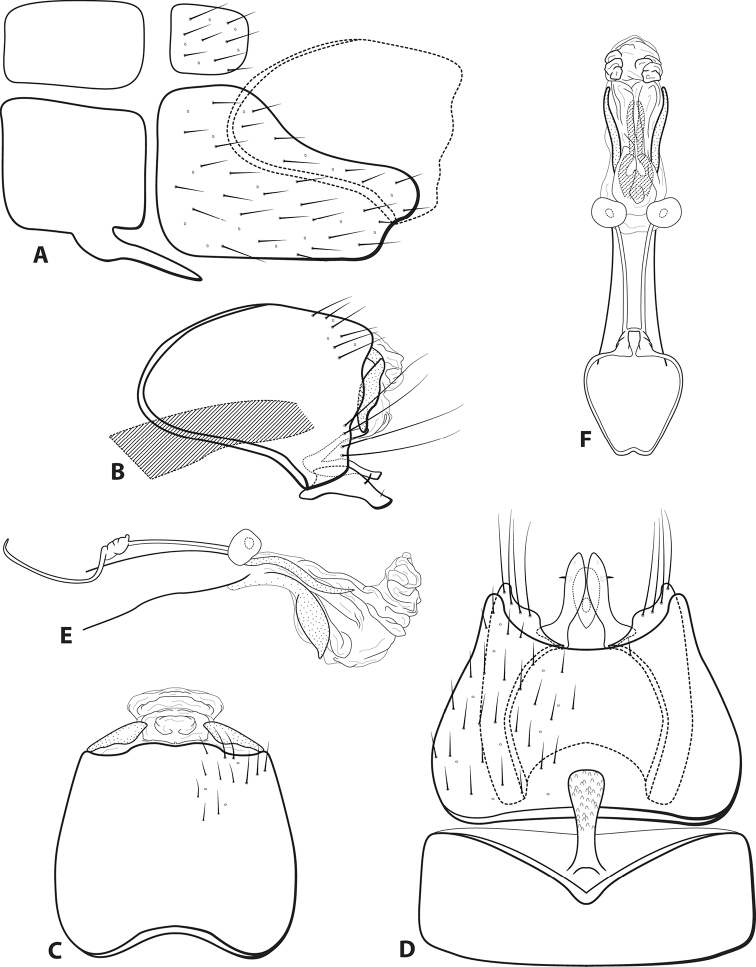
*Leucotrichiaviridis* Flint, 1967. Male genitalia: **A** segments VII–VIII and segment IX margin, lateral **B** segments IX–X, lateral (base of phallus crosshatched) **C** segments IX–X, dorsal **D** segments VII–IX, ventral **E** phallus, lateral **F** phallus, dorsal. Modified from Thomson and Holzenthal (2015).

###### Material examined.

**Panama: Bocas del Toro Province** • 21 males; Cuenca 093, Chiriqui Grande District, Quebrada Rambala, Rambala Jungle Lodge; 8.91627°N, 82.15469°W; 120 m a.s.l.; 9 Aug. 2014; E. Carlson, leg.; Malaise trap; in alcohol; MUPADI • ibid., 3 males; 28 Mar. 2015; UV light trap • ibid., 3 males; 31 Mar.–11 Apr. 2015; Malaise trap. **Chiriqui Province** • 1 male; Cuenca 108; Dolega District; Río Majagua; Potrerillos, Banquito de Palmira; 8.68083°N, 82.53253°W; 840 m a.s.l.; 28 Feb.–14 Mar. 2019; T. Ríos, Y. Aguirre, leg.; Malaise trap; in alcohol; MUPADI. –**Veraguas Province** • 3 males; Cuenca 132; Santa Fe District, Santa Fe National Park, Río Mulaba, afl. 1er Brazo; PSPSCB-NPSF-C-097-2017-008; 8.51706°N, 81.1214°W; 770 m a.s.l.; E. Álvarez, E. Pérez, T. Ríos, leg.; 19–23 Apr. 2017; in alcohol; COZEM.

###### Distribution.

El Salvador, Guatemala, Mexico, Panama.

#### *Leucotrichapictipes* group

##### ﻿ Leucotrichia fairchildi

Taxon classificationAnimaliaTrichopteraHydroptilidae

Flint, 1970

1CF515C5-ACD3-53BE-A330-D44C7DECF5C4

Figs 2C , 16

###### Diagnosis.

*Leucotrichiafairchildi* is currently the only member of the *L.pictipes* Group recorded in Panama. *Leucotrichiapictipes* known distribution includes Mexico, while *L.imitator* Flint, 1970 and *L.sarita* Ross, 1944 have been recorded in nearby Costa Rica. All species in this group bear only two ocelli and share similar shapes to the inferior appendage and sternum VII, when viewed laterally. The modified basal antennal segments and setiferous production on the dorsum of the head can be used to easily separate *L.fairchildi* from each of the three other species.

**Figure 16.**
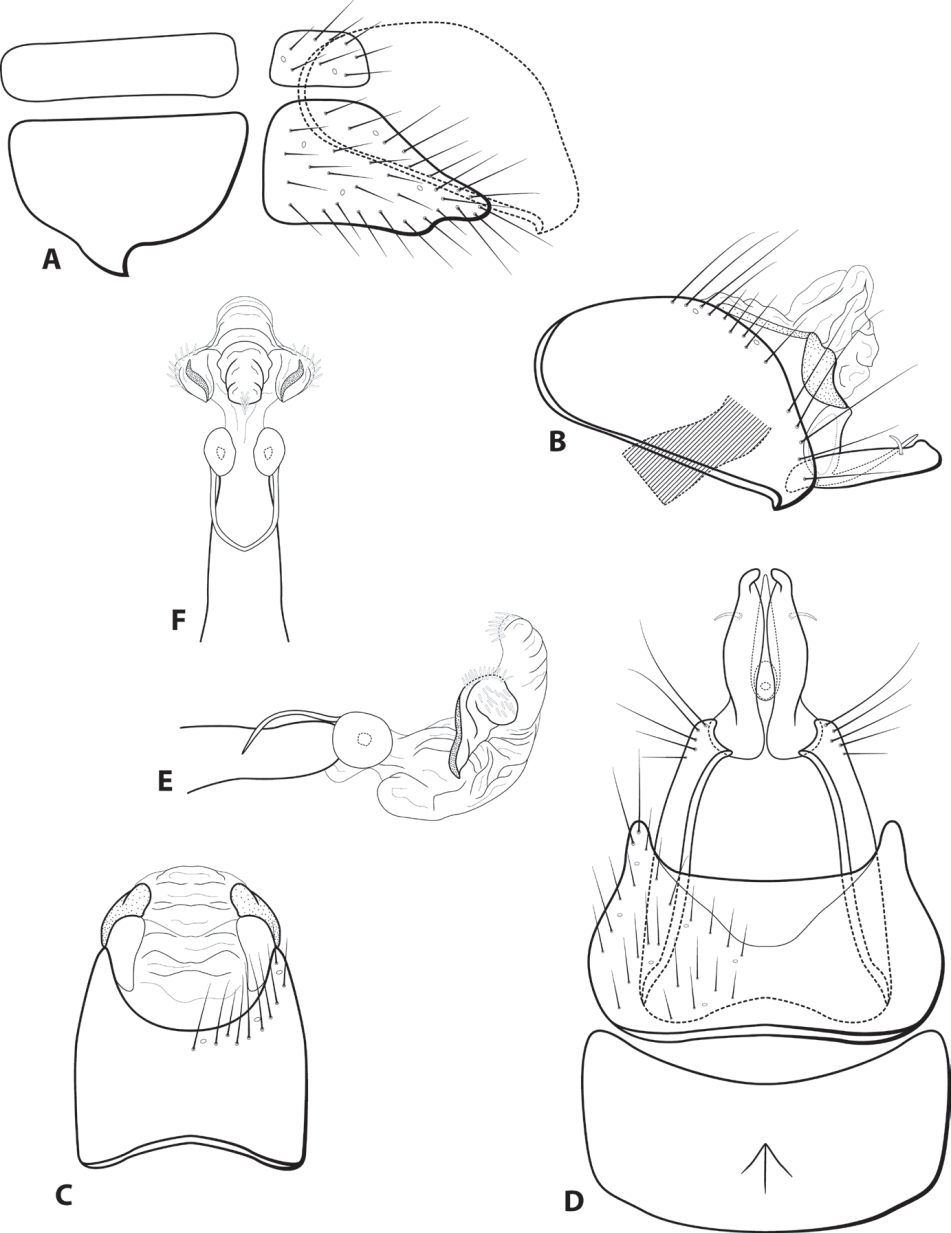
*Leucotrichiafairchildi* Flint, 1970. Male genitalia: **A** segments VII–VIII and segment IX margin, lateral **B** segments IX–X, lateral (base of phallus crosshatched) **C** segments IX–X, dorsal **D** segments VII–IX, ventral **E** phallus, lateral **F** phallus, dorsal. Modified from Thomson and Holzenthal (2015).

###### Material examined.

Panama: **Bocas del Toro Province** • 16 males; Cuenca 093; Chiriqui Grande District; Quebrada Rambala; Rambala Jungle Lodge; 8.91627°N, 82.15469°W; 120 m a.s.l.; 21–31 Dec. 2016; E. Carlson, leg.; Malaise trap; in alcohol; MUPADI • ibid., 3 males; 6–12 Feb. 2017 • ibid., 1 male; 12–15 Jun. 2017 • 2 males; 28–30 Jun. 2017. **Comarca Ngäbe Buglé** • 3 males; Quebrada Martinez, Bosque Protector Palo Seco, Alto de Valle, detrás de las caseta de MiAmbiente; 8.79484°N, 82.19391°W, 480 m a.s.l.; 16–30 Aug. 2019; Y. Aguirre, T. Ríos, leg.; Malaise trap; in alcohol; MUPADI • ibid., 2 males; 30 Aug.–13 Sep. 2019 • ibid., 2 males; 13–27 Sep. 2019. **Veraguas Province** • 1 male; Cuenca 097; Santa Fe District; Santa Fe National Park; Río Piedra de Moler; PSPSCB-NPSF-C-097-2017-011; 8.55343°N, 81.17675°W; 395 m a.s.l.; 20 Apr. 2017; T. Ríos, E. Álvarez, C. Nieto, leg.; UV light trap; in alcohol; COZEM.

###### Distribution.

Colombia, Costa Rica, Ecuador, El Salvador, Grenada, Panama, Tobago, Trinidad, Venezuela.

###### Remarks.

A pharate female in a pupal case was identified by Botosaneanu and Alkins-Koo (1993) as “Leucotrichiini—case 2”. Subsequently, Flint (1996) associated this female with *L.fairchildi*, in addition to collecting adult material from Trinidad and Venezuela.

## ﻿Discussion

### ﻿*Leucotrichia* – A mystery wrapped in an enigma

The colloquial expression “I know it when I see it” was coined in 1964 by United States Supreme Court Justice Potter Stewart to describe his threshold test for obscenity in Jacobellis v. Ohio. As stated in the Results section, a definitive diagnosis for adults of the genus *Leucotrichia* is difficult at this time and could only be objectively based on one or perhaps two characters. But, subjectively it is more easily perceived. And, to support this perception, this position is confirmed by molecular analyses (Santos et al. 2016). This perception was successfully applied to each species included in the Results section in the process of producing this assemblage of *Leucotrichia* species for Panama.

**Figure 17.**
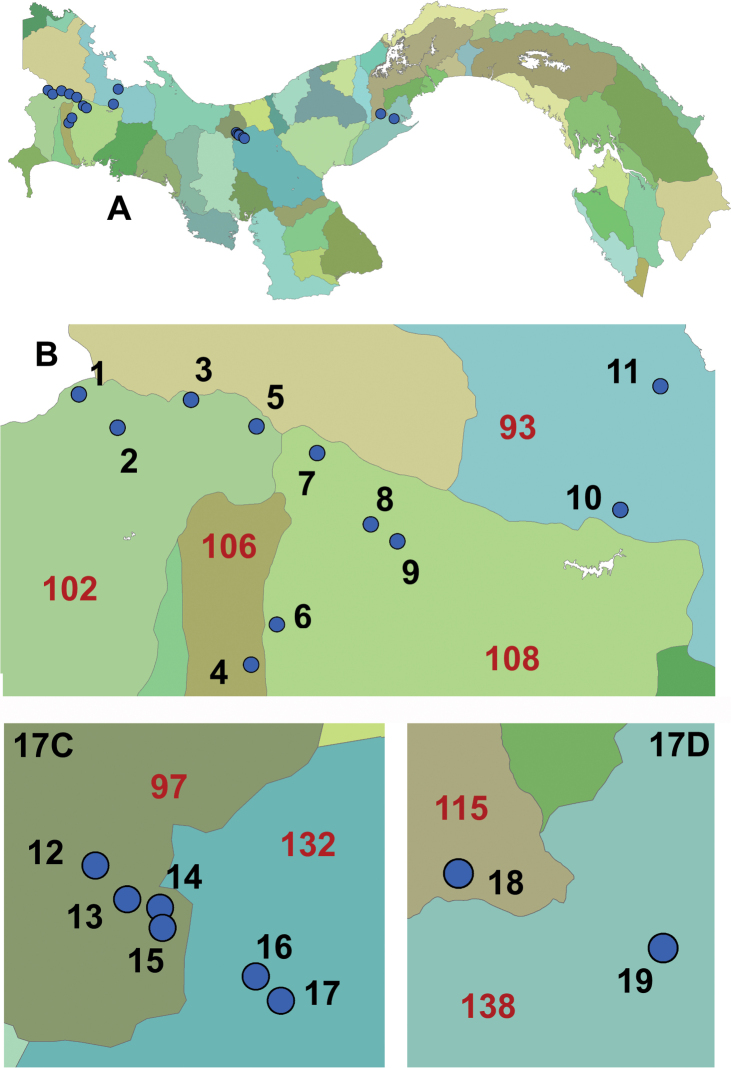
Locations of collection sites for *Leucotrichia* in Panama, sorted and displayed by longitude, then latitude **A** map of Panama showing collection locations **B** enlarged map of western Panama showing locations 1–11 **C** map of the Santa Fe National Park locations12–17 **D** map of the Altos de Campana National Park locations 18 and 19. Please refer to Tables 1 and 2 for additional information.

In the course of our studies in Panama, we have added to the confusion by finding several species which are “exceptions to the rule” for characters which normally would be included in a diagnosis for the genus. For example, unmodified wings were a consistent, albeit unremarkable, character for this genus, until we described *L.ruiteri* in this paper. This new species has a forewing which bears a pocket filled with scales.

Another, formerly reliable character typical of *Leucotrichia* is the subgenital plate with dorsal and ventral arms. However, a survey of the genus shows that only 21 species (e.g., the Panamanian species *L.botosaneanui*) have both arms on the subgenital plate. A total of 21 other species (e.g., the Panamanian species *L.melleopicta*) lacks the dorsal arm. And, four species (*L.adela*, *L.inflaticornis*, *L.laposka* Oláh & Johanson, 2011, *L.tubifex* Flint, 1964) are undetermined at this time.

Other characters which could have been used in a diagnosis vary by number or presence. For example, two ocelli are characteristic of the *L.pictipes* group and three ocelli are found in all members of the *L.melleopicta* Group. Inferior appendage segments are fused in 15 species (e.g., the Panamanian species, *L.rhomba*), with separate segments in 29 species (e.g., the Panamanian species *L.chiriquiensis*), and undetermined in two species (*L.adela*, *L.alisensis* Rueda Martín, 2011). There is a dorsal spine on the inferior appendage in 35 species (e.g., *L.melleopicta*), it is lacking in ten species (e.g., *L.inops* Flint, 1991), and is undetermined in one species (*L.adela*). Forty-one species have a ventral process on abdominal segment VII (prominent in 33 species, e.g., *L.melleopicta*; reduced in eight species, e.g., *L.botosaneanui*), and six species have no ventral process.

Finally, there are other characters, like the unmodified wings mentioned above, which seem consistent within *Leucotrichia*, but are shared with other genera. An example of this is the median complex on the phallus bearing sclerotized armature, which is also found in *Zumatrichia* and other members of the Leucotrichiinae.

Of course, the variability that we find within this genus is also common in other genera of insects. However, usually there is a core of morphological characters which consistently define those genera. Even so, in Santos et al. (2016), there was only a single morphological character in the larvae which united the genus. At this juncture, the best we can say is that *Leucotrichia* is a genus consisting of a complex of species, united by perception and supported by molecular analyses, not all of whom share all morphological characters consistently. Future molecular and morphological analyses could alter our definition of this genus, further clarifying its position and uniqueness, or lack thereof, within the Leucotrichiinae. However, until then we are confident that “we know it when we see it”.

### ﻿How many *Leucotrichia*?

When we first started examining the extent of the hydroptilid fauna of Panama in 2015, we were naively comfortable with the three taxa representing the genus *Leucotrichia*. Two of them (*L.chiriquiensis* and *L.fairchildi*) had been described from this country, and the thought that many more were undetected seemed remote. This presumption was supported by recent papers based on two doctoral studies involving this genus (Thomson and Holzenthal 2015; Santos et al. 2016), neither of which identified new taxa for Panama. Our calm was somewhat disturbed during subsequent years as we began to find new first records for *Leucotrichia* as the result of prolonged sampling at single locations, involving Malaise trapping. Prior to 2012, almost all caddisflies collected and identified from Panama resulted from light trapping or sweeping. We began to see other possibilities in 2020 when we detected our first new species from Panama, *L.cultrata* (Thomson and Armitage 2021). However, we were totally unprepared, as we began to address unidentified leucotrichiine specimens in preparation for generating this manuscript, to find we had four additional new species to science and two additional first records for Panama. Where would it end? We now have replaced complacency with anticipation as we collect, process, and identify each new sample. This anticipation is further supported by the fact that Panama’s assemblage of *Leucotrichia* species has a low similarity to those found in neighboring countries (see below). More new species to science are possible, while the chance for more first records for Panama seems probable.

The 14 species of this genus in Panama is currently the most for any Latin American country. Impressive as that total is, we must dampen our enthusiasm in at least two regards. Latin America as a whole is considerably under-collected for adult caddisflies. Whereas a number of countries have a published species list (e.g., Bueno-Soria and Flint (1978) for Mexico, Chamorro-Lacayo et al. (2007) for Nicaragua, Holzenthal (1988) for Costa Rica, Muñoz-Quesada (2000) for Colombia, Ríos-Touma et al. (2017) for Ecuador, and Paprocki and França (2014) and Santos et al. (2020) for Brazil), they all are but intermediate waypoints. All imply room for growth. For example, Ríos-Touma et al. (2017) listed 310 species for Ecuador, but based on a non-parametric estimator of true species richness (Chao2; Shen et al. 2003; Gotelli and Colwell 2011), this represents only ~ 54% of its estimated species richness. In Panama, we are quickly approaching 500 total species, with no end in sight; and this for a country ~ 28% the size of Ecuador and with less topographic diversity. In addition, little of the collecting which has taken place in these other countries involved Malaise traps, which we consider a critical factor. As more collecting in other countries takes place, involving prolonged sampling with multiple methods, we anticipate that the *Leucotrichia* assemblages of those countries will increase significantly, and perhaps exceed what we find in Panama.

### ﻿Coexistence and frequency of occurrence

It is not uncommon to find multiple congeners of many caddisfly genera in the same stream location. Intuitively, there are sufficient resources available to mitigate any possible competition or cropping by predators to keep population levels relatively low. Thus, the competitive exclusion priniciple (aka Gause’s Law; Gause 1932), in most cases, does not apply, particularly for microcaddisflies who cannot be imagined to impact resource levels in all but the most narrow of niches. However, as noted in Table 3, there are on average, roughly three species for each of the 19 streams in which *Leucotrichia* have been found in Panama. The actual tallies can be found in Table 1, and the range is considerable. Two of the streams, Quebrada Jaramillo (1,214 m a.s.l.) and Quebrada Rambala (120 m a.s.l.), both in dendritic watersheds, each have seven species of *Leucotrichia* which have been collected at the same locations. The Río Majagua (840 m a.s.l.), which is a linear watershed coming off of Volcan Baru, has produced six species to date, with identifications on-going. Most of the other streams listed in Table 1 have four or fewer species. Considering that the three streams mentioned above were sampled with both UV light and Malaise traps monthly for at least a year, one might suspect that the other 16 streams have additional species to reveal if only they were sampled more thoroughly. Regardless, these few examples demonstrate a species packing that exceeds expectations. Whether the small size of these species facilitates the packed nature of their assemblages remains to be determined. This is another pointed example of the need for prolonged sampling with multiple methods to better define caddisfly assemblages at any location.

### ﻿Altitudinal distribution

**Figure 18.**
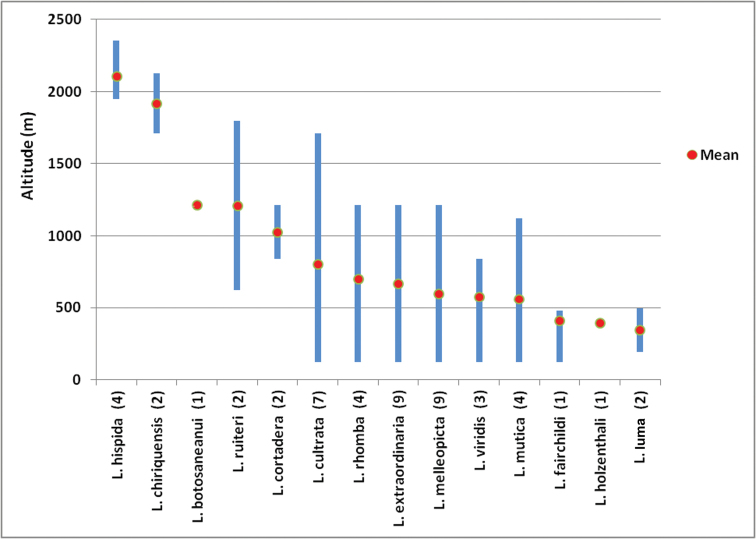
Distribution of *Leucotrichia* species with altitude in Panama, sorted from left to right by mean values. The number of unique streams involved in plotting each species is found in parentheses following each species name. Mean values for each species distribution range are indicated by a red circle. The species name labels orthogonal to the X-axis are not italicized to facilitate readability.

There are a number of problems when evaluating the distribution of any group of aquatic insects in relation to altitude. Inadequate and infrequent sampling at representative altitudes, rarely captured or undetected species, low or high altitude outliers caused by meteorological events, differences in stream velocity and riparian corridor composition, and meteorological conditions during sampling are but a few (Janzen 1967; Rahbek 1995; Miserendino and Pizzolón 2001). With those caveats exposed, we present the distribution of *Leucotrichia* species with altitude in Fig. 18. It is apparent that most of the species can be found from low (~ 100 m a.s.l.) to middle (~ 1,500 m a.s.l.) altitudes. The higher altitudes appear to be populated by very few species consistently, while another few species have only been collected at low altitudes. Approximately 1/3 of the species in Panama are infrequently collected (Table 3), including those that favor low altitudes, so until additional specimens of these species are collected, we reserve judgment about their true range of altitudes occupied. However, we think that sufficient collections have been made overall to make us more confident about the higher altitude proclivities of *L.hispida* and *L.chiriquiensis*. Additional data records from monthly Malaise trap samples over several years bolster our belief in these two species being confined to higher altitudes.

### ﻿Geographic distributions and affinities

Endemics aside, the current, typical distribution pattern among caddisflies in the northern Neotropics follows a NW to SE axis from Mexico down to Panama, with disjuncts to the north (nearctic North America) and to the south (northern South America). Most of the species of *Leucotrichia* in Panama follow this track. However, there are two species of *Leucotrichia* and roughly two dozen Panamanian caddisfly species which share the Trinidad to Panama connection mentioned in the Results section. To our knowledge, few other northern South American countries between Trinidad and Panama also host these species. We have speculated in the past that the higher velocity trade winds during the dry season (December through April) moving from northern South America west, toward and across Panama, might be involved in this disjunct distribution (Armitage et al. 2020). However, other than recording yet more species which fit this profile, no other additional proof has been obtained.

*Leucotrichia* species have been found in 22 continental and island countries in Latin America, including the Caribbean Region (Holzenthal and Calor 2017). Surprisingly, none have been found in French Guiana, Suriname, Paraguay, Uruguay, Bolivia, Cuba, Belize, Honduras, and many of the Caribbean Islands. We suspect, in large part, this absence is the result of undercollecting of adults. Also, the majority of our species were collected in Malaise traps, whose use is important for detecting the greatest number of species for this genus at a collecting site, but not commonly employed elsewhere. The lack of affinity among countries for this and other genera appears negatively correlated with the percent of endemics in each country’s fauna. In samples from Panama, we have observed from 20–35% endemics. In Brazil (Santos et al. 2020), 538 of the 796 recorded species of caddisflies are endemic (68%). We suggest that as more collecting is done, the affinity among countries will increase and the percent of endemics in each country will go down. We base this statement, in part, on our elimination of “endemic” status from 84 Costa Rican species of caddisflies over the last six years through their discovery in Panama.

An interesting historical aspect of this puzzling genus involves its ancestral home or point of origin and the genesis of its current assemblage of species. If we accept the work of Santos et al. (2016), based on molecular analyses, the Leucotrichiinae began to diversify some 124 ma, after the separation of South America from Africa. Crown diversification of the Leucotrichiini occurred ~ 80 ma. As South America assumed its current orientation and position, they proposed that the Leucotrichiini or its generic derivatives migrated north, using the proto-Caribbean archipelago as an initial invasion corridor. This is consistent with the theory that Brazil and the Amazon basin are the center of origin or ancestral home for many organismal groups (Antonelli et al. 2018). As mentioned above, the genus *Leucotrichia* in Brazil is at the moment characterized by low diversity and no affinity to other countries in Latin America, and there is the possibility that the genus evolved elsewhere. However, given that all other Leucotrichiini genera are present in South America, with some restricted to this region, it is more plausible that the genus evolved in South America and then dispersed northward (A. Santos, pers. comm.). The only other hard information we have is based on the work of Wells and Wichard (1989) wherein they described the fossil species *Leucotrichiaadela* from Dominican amber (20–23 ma), diagnosing it as closest to the extant Panamanian species, *L.chiriquiensis*. Based on all of this, there is a reasonable likelihood that the original migrants from South American initially diversified and underwent rapid radiation in Caribbean and Central America regions, not South America, as the next step in producing the array of species we have today. Then alternating changes in climate during the Pleistocene, and before, with reciprocating northward and southward migrations of floras and faunas (Rocha and Kaefer 2019), could have forced additional speciation, as well as colonization of northern South America by some species which evolved further north. All of this is conjecture, of course, and our understanding of the matter will only become clearer with increased collections and with more geographically inclusive molecular work on the leucotrichiine genera and species.

**Figure 19.**
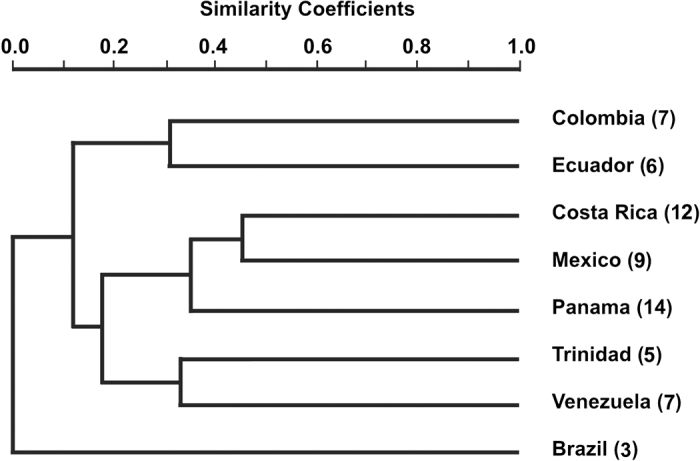
Cluster diagram employing Bray-Curtis similarity values showing the relationships between Latin American countries home to at least four species of *Leucotrichia*, plus Brazil. The number of species in each country is indicated within parentheses at the end of each name.

The composition and structure of the cluster diagram in Fig. 19 will surely change as more sampling and identifications of adult *Leucotrichia* takes place in all Latin American countries. We anticipate a marked increase of similarity values across the board, should that occur. This low similarity between Panama and other Latin American countries (Fig. 19), also increases the probability that other species, but certainly not all, from outside Panama will eventually be found here. This is based on more than presumption. The majority of the new country records (*n* = 156) we have found during the last six years are species which heretofore were only found in Costa Rica. Thus, other *Leucotrichia* species unique to that country have a higher probability of being found in Panama. This same logic holds, to a lesser degree, with other countries with which Panama currently shares *Leucotrichia* species.

## Supplementary Material

XML Treatment for Leucotrichia botosaneanui

XML Treatment for Leucotrichia chiriquiensis

XML Treatment for Leucotrichia cortadera

XML Treatment for Leucotrichia cultrata

XML Treatment for Leucotrichia extraordinaria

XML Treatment for Leucotrichia hispida

XML Treatment for Leucotrichia holzenthali

XML Treatment for Leucotrichia luma

XML Treatment for Leucotrichia melleopicta

XML Treatment for Leucotrichia mutica

XML Treatment for Leucotrichia rhomba

XML Treatment for Leucotrichia ruiteri

XML Treatment for Leucotrichia viridis

XML Treatment for Leucotrichia fairchildi

## References

[B1] Antonelli A Zizka A Carvalho FA Scharna R Bacon CD Silvestro D Condamineb FL (2018) Amazonia is the primary source of Neotropical biodiversity. Proceedings of the National Academy of Sciences of the United States of America 115: 6034–6039. 10.1073/pnas.1713819115 29760058 PMC6003360

[B2] Armitage BJ Blahnik RJ Harris SC Cornejo A Arefina-Armitage TI (2018) The Trichoptera of Panama. VII. Additional new country records for caddisflies from the Republic of Panama. Insecta Mundi 0614: 1–7.

[B3] Armitage BJ Harris SC Blahnik RJ Thomson RE (2016) The Trichoptera of Panama IV. New records for caddisflies (Insecta: Trichoptera) from the Republic of Panama. Insecta Mundi 0511: 1–13.

[B4] Armitage BJ Harris SC Blahnik RJ Thomson RE Ríos TA Aguirre Y (2020) The Trichoptera of Panama XIII. Further new country records for caddisflies (Insecta: Trichoptera) from the Republic of Panama. Insecta Mundi 0744: 1–8.

[B5] Blahnik RJ Holzenthal RW Prather AL (2007) The lactic acid method for clearing Trichoptera genitalia. In: Bueno-Soria J Barba-Álvarez R Armitage BJ (Eds) Proceedings of the 12th International Symposium on Trichoptera.The Caddis Press, Columbus, Ohio, 9–14.

[B6] Bueno-Soria J Flint Jr OS (1978) Catálogo sistemático de los tricopteros de México (Insecta: Trichoptera), con algunos registros de Norte, Centro y Sudamérica. Anales del Instituto de Biología, Universidad Nacional Autónoma de México, Serie Zoología 49: 189–218.

[B7] Calor AR Mariano R (2012) UV light pan traps for collecting aquatic insects. EntomoBrasilis 5: 164–166. 10.12741/ebrasilis.v5i2.187

[B8] Chamorro-Lacayo ML Maes J-M Holzenthal RW Blahnik RJ (2007) Updated checklist of the Trichoptera of Nicaragua. In: Bueno-Soria J Barba-Álvarez R Armitage BJ (Eds) Proceedings of the 12th International Symposium on Trichoptera.The Caddis Press, Columbus, Ohio, 37–50.

[B9] Dallwitz MJ Paine TA Zurcher EJ (2016) User’s guide to the DELTA Editor. http://delta-intkey.com/www/delta-ed.htm [Last accessed October 2017]

[B10] Flint Jr OS (1970) Studies of Neotropical caddisflies, X: *Leucotrichia* and related genera from North and Central America (Trichoptera: Hydroptilidae). Smithsonian Contributions to Zoology 60: 1–64. 10.5479/si.00810282.60

[B11] Gause GF (1932) Experimental studies on the struggle for existence: 1. Mixed population of two species of yeast. Journal of Experimental Biology 9: 389–402. 10.1242/jeb.9.4.389

[B12] Gotelli NJ Colwell RK (2011) Estimating species richness. Biological Diversity: Frontiers in Measurement and Assessment 12: 39–54.

[B13] Harris SC Armitage BJ (2019) The Trichoptera of Panama. X. The Quebrada Rambala Drainage, with description of 19 new species of microcaddisfies (Trichoptera: Hydroptilidae). Insecta Mundi 0707: 1–54.

[B14] Holzenthal RW (1988) Catalogo systematico de los Trichopteros de Costa Rica (Insecta: Trichoptera). Brenesia 29: 51–82.

[B15] Holzenthal RW Calor AR (2017) Catalog of the Neotropical Trichoptera (Caddisﬂies). ZooKeys 654: 1–566. 10.3897/zookeys.654.9516 PMC5345355 28331396

[B16] Janzen DH (1967) Why mountain passes are higher in the tropics. The American Naturalist 101: 233–249. 10.1086/282487

[B17] Marshall JE (1979) A review of the genera of the Hydroptilidae (Trichoptera). Bulletin of the British Museum (Natural History) Entomology 39(3): 135–239.

[B18] Miserendino ML Pizzolón LA (2001) Abundance and altitudinal distribution of Ephemeroptera in an Andean-Patagonean river system (Argentina). In: Dominguez E (Ed.) Trends in research in Ephemeroptera and Plecoptera.Proceedings of the IX^th^ International Conference on Ephemeroptera and XIII^th^ International Symposium on Plecoptera. Kluwer Academic/Plenum Press, New York, New York, 135–142. 10.1007/978-1-4615-1257-8_16

[B19] Muñoz-Quesada F (2000) Especies del orden Trichoptera (Insecta) en Colombia. Biota Colombiana 1: 267–288.

[B20] Oláh J Johanson KA (2011) New Neotropical Hydroptilidae (Trichoptera). Annales Histori­co-Naturales Musei Nationalis Hungarici 103: 117–255.

[B21] Paprocki H França D (2014) Brazilian Trichoptera Checklist II. Biodiversity Data Journal 2: e1557: 1–109. 10.3897/BDJ.2.e1557 PMC4206778 25349524

[B22] Rahbek C (1995) The elevational gradient of species richness: a uniform pattern? Ecography 18: 200–205. 10.1111/j.1600-0587.1995.tb00341.x

[B23] Ríos-Touma B Holzenthal RW Huisman J Thomson RE Rázuri-Gonzales E (2017) Diversity and distribution of the Caddisflies (Insecta: Trichoptera) of Ecuador. PeerJ 5: e2851. 10.7717/peerj.2851 PMC5237369 28097062

[B24] Rocha DG Kaefer IL (2019) What has become of the refugia hypothesis to explain biological diversity in Amazonia? Ecology and Evolution 9(7): 4302–4309. 10.1002/ece3.5051 PMC6468052 31016006

[B25] Santos APM Nessimian JL Takyia DM (2016) Revised classification and evolution of leuchotrichiine microcaddisflies (Trichoptera: Hydroptilidae) based on morphological and molecular data. Systematic Entomology 41: 458–480. 10.1111/syen.12168

[B26] Santos APM Dumas LL Henriques-Oliveira AL Souza WRM Camargos LM Calor AR Pes AMO (2020) Taxonomic Catalog of the Brazilian Fauna: order Trichoptera (Insecta), diversity and distribution. Zoologia 37: e46392. 10.3897/zoologia.37.e46392

[B27] Shen TJ Chao A Lin CF (2003) Predicting the number of new species in further taxonomic sampling. Ecology 84(3): 798–804. 10.1890/0012-9658(2003)084[0798:PTNONS]2.0.CO;2

[B28] Thomson RE Armitage BJ (2021) The Trichoptera of Panama. XV. Six new species and four new country records of microcaddisflies (Insecta: Trichoptera: Hydroptilidae) from Mount Totumas Cloud Forest and Biological Reserve. Revista Mexicana de Biodiversidad 92: e923631. 10.22201/ib.20078706e.2021.92.3631

[B29] Thomson RE Holzenthal RW (2012) New species and records of Hydroptilidae (Trichoptera) from Venezuela. ZooKeys 185: 19–39. 10.3897/zookeys.185.2909 PMC3345792 22577311

[B30] Thomson RE Holzenthal RW (2015) A revision of the Neotropical caddisfly genus *Leucotrichia* Mosely, 1934 (Hydroptilidae: Leucotrichiinae). ZooKeys 499: 1–100. 10.3897/zookeys.499.8360 PMC4410161 25931968

[B31] Wells A Wichard W (1989) Caddisflies of Dominican amber VI. Hydroptilidae (Trichoptera). Studies in Neotropical Fauna and Environment 24: 41–51. 10.1080/01650528909360774

[B32] Wiggins GB (1996a) Larvae of the North American Caddisfly Genera (Trichoptera), 2^nd^ edn. University of Toronto Press, Toronto, Canada, 457 pp. 10.3138/9781442623606

[B33] Wiggins GB (1996b) Chapter 17: Trichoptera Families. In: Merritt RW Cummins KW (Eds) An Introduction to the Aquatic Insects of North America (3rd edn., revised). Kendall/Hunt, Dubuque, Iowa, 309–349.

